# Investigations into the antibacterial effects and potential mechanism of gambogic acid and neogambogic acid

**DOI:** 10.3389/fmicb.2022.1045291

**Published:** 2022-12-12

**Authors:** Mingzhu Li, Yuan Chen, Lijuan Wang, Chujie Lu, Peiying Chen, Yuanling Jin, Jiacong Li, Fei Gao, Zhuo Shang, Wei Lin

**Affiliations:** ^1^Jiangsu Collaborative Innovation Center of Chinese Medicinal Resources Industrialization, Nanjing, China; ^2^Department of Pathogen Biology, School of Medicine and Holistic Integrative Medicine, Nanjing University of Chinese Medicine, Nanjing, China; ^3^School of Pharmacy, Nanjing University of Chinese Medicine, Nanjing, China; ^4^School of Pharmaeutical Sciences, Shandong University, Jinan, China; ^5^State Key Laboratory of Drug Research, Shanghai Institute of Materia Medica, Chinese Academy of Sciences, Nanjing, China; ^6^State Key Laboratory of Microbial Resources, Institute of Microbiology, Chinese Academy of Sciences, Nanjing, China

**Keywords:** antibiotic resistance, *Enterococcus faecalis*, gambogic acid, neogambogic acid, undecaprenyl pyrophosphate synthase, infection

## Abstract

The growing threat of antibiotic-resistant bacterial infections to public health necessitates the development of novel antibacterial agents. Inhibiting bacterial cell wall synthesis has remained a key focus for antibiotic development. Our search for inhibitors of undecaprenyl diphosphate synthase (UPPS), an essential enzyme required for bacterial cell wall formation, revealed that two primary components of gamboge, gambogic acid (GA) and neogambogic acid (NGA), significantly inhibited the activity of *Enterococcus faecalis* UPPS (*Efa*UPPS) with the half maximal inhibitory concentrations (IC_50_) of 3.08 μM and 3.07 μM, respectively. In the *in vitro* antibacterial assay, both GA and NGA also exhibited inhibitory activities against *E. faecalis* with the minimal inhibitory concentrations (MICs) of 2 μg/mL. Using microscale thermophoresis, molecular docking, and enzymatic assays, we further confirmed that GA and NGA occupy the substrate binding pocket of *Efa*UPPS with micro-molar binding affinity, preventing the natural substrates farnesyl diphosphate (FPP) from entering. Mutagenesis analysis revealed that L91 and L146 are two key residues in the binding between GA/NGA and UPPS. Furthermore, we also demonstrated that GA and NGA can improve *E. faecalis*-induced undesirable inflammation in a mouse infection model. Taken together, our findings provide a basis for structural optimization of GA/NGA to develop improved antibiotic leads and enhance treatment success rates in clinical practice.

## Introduction

The emergence of antibiotic-resistant bacterial infections poses a serious threat to public health. However, the overuse and abuse of existing antibiotics as well as diminishing antibiotic pipelines have resulted in concerning levels of bacterial multidrug resistance and loss of the therapeutic efficacy of many antibiotics (Laxminarayan et al., 2013; Yoshida et al., 2017). Emerging evidence suggests that some drug-resistant bacteria, such as vancomycin-resistant *Enterococci* (VRE) and methicillin-resistant *Staphylococcus aureus* (MRSA), have also developed resistance to many other antibiotics (Hiramatsu et al., 1997; Raza et al., 2018; Miller et al., 2020), but there are few antibiotics to combat these multidrug resistance bacteria. As a result, there is an undeniable need for the development of novel antibiotics with new targets, as well as the rational optimization and remodeling of existing anti-infective agent scaffolds to aid in the fight against drug resistance.

Bacterial cell wall has long been an interesting target for antibiotic discovery (Schneider and Sahl, 2010; Williams et al., 2020). Peptidoglycan, a polymer of peptide and glycan strands cross-links, is the major component of cell wall structure (Vollmer et al., 2008; Turner et al., 2014; Dorr et al., 2019). Most commercial antibiotics that inhibit bacterial cell wall synthesis, such as penicillin (Park and Strominger, 1957), vancomycin (Perkins, 1969) and bacitracin (Storm and Strominger, 1973), target the late stage of peptidoglycan formation. Antibiotics that target the early stage, on the other hand, have received little attention. Early steps of bacterial cell wall biosynthesis involve the condensation of dimethylallyl diphosphate (DMAPP) with two molecules of isopentenyl diphosphate (IPP) to generate farnesyl diphosphate (FPP) catalyzed by FPP synthase (FPPS). The resulting FPP further condenses with eight additional IPP molecules to afford undecaprenyl diphosphate (UPP) catalyzed by UPP synthase (UPPS). Dephosphorylation of UPP produces undecaprenyl phosphate (UP), which is a key lipid involved in the biosynthesis of peptidoglycan and many other cell-wall polysaccharide (Jomaa et al., 1999; Scholte et al., 2004; Guo et al., 2005; Teng and Liang, 2012; Inokoshi et al., 2016; Wang et al., 2016; Dhar et al., 2018; Vollmer et al., 2019). In this regard, UPPS plays an important role in the early stages of bacterial cell wall synthesis, making it a prospective target for antibiotic discovery (Teng and Liang, 2012; Inokoshi et al., 2016). To date, only several synthetic UPPS inhibitors have been reported (Kuo et al., 2008; Teng and Liang, 2012; Wang et al., 2016). Therefore, discovery of UPPS inhibitors is an effective strategy for new antibiotics combating MRSA, VRE, and other drug-resistant bacteria.

**Figure 1:**
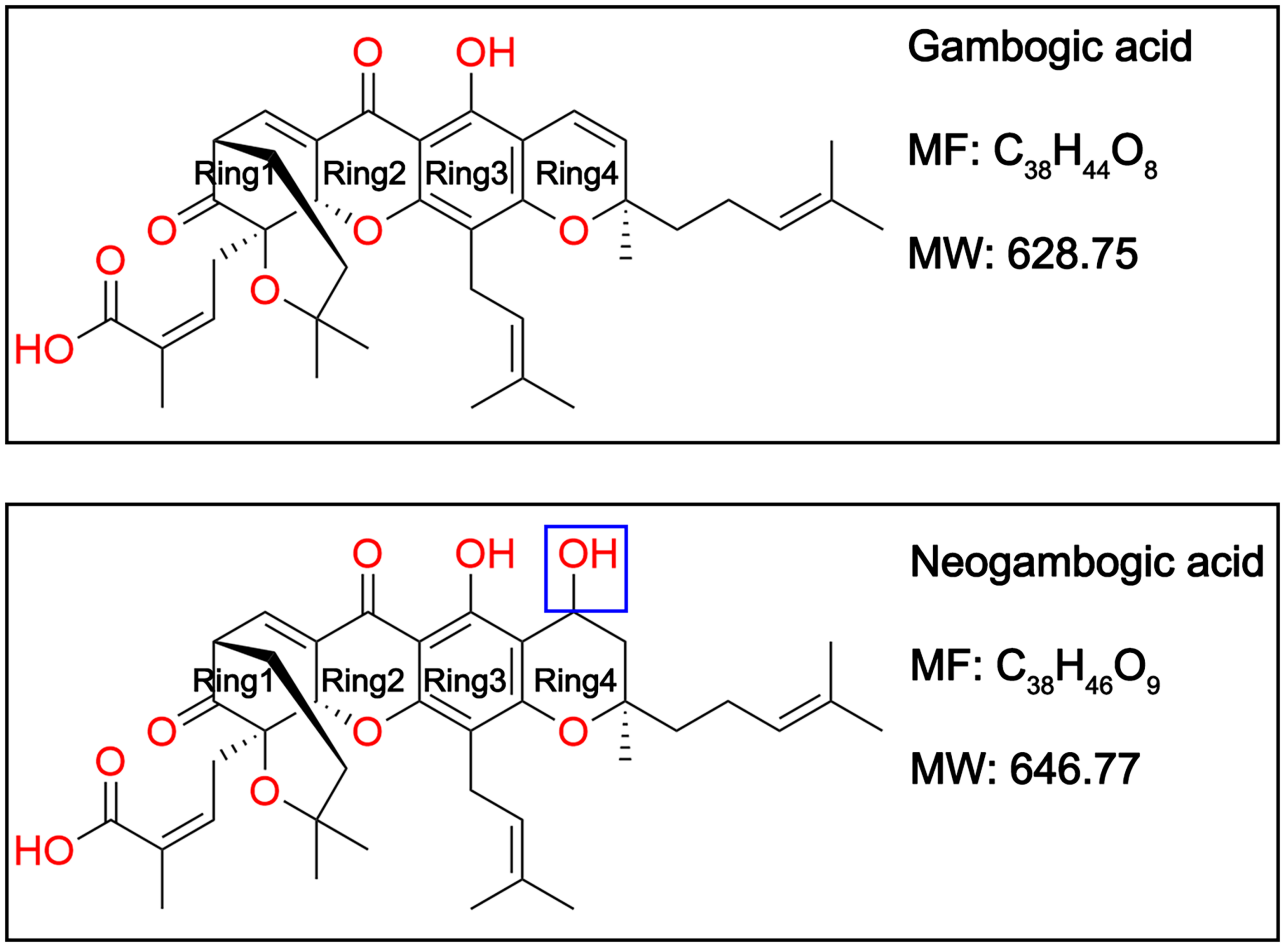
Chemical structures of gambogic acid (GA) and neogambogic acid (NGA).

Plant natural products have been an excellent source of drugs (Atanasov et al., 2021), which is exemplified by the anti-inflammatory agent acetylsalicylic acid (aspirin) derived from the natural product salicin isolated from *Salix alba* L (Dias et al., 2012; Montinari et al., 2019). Gambogic acid (GA) and neogambogic acid (NGA, also named gambogenic acid) are the major bioactive components of gamboge, a dry resin derived from the traditional Chinese medicine *Garcinia hanburyi* (Asano et al., 1996; Deng et al., 2012). Structurally, GA is a caged prenylated garcinia xanthone whereas NGA is a C3-C4 hydration form of GA (Weakley et al., 2001; Han et al., 2005; Figure 1). A number of studies has reported that GA and NGA display promising anti-cancer effects towards different types of cancer cell lines (Wu et al., 2004; Zhao et al., 2004; Li et al., 2010; Wang and Chen, 2012; Yu et al., 2012; Zhou et al., 2020). Moreover, GA has been certified by the China Food and Drug Administration for clinical trials (Chi et al., 2013; Yang and Chen, 2013). However, there are limited number of reports documenting antibacterial activities of GA and NGA. Although Chaiyakunvat and Hua *et al* have reported that GA and NGA possess potential antibacterial activity against MRSA (Chaiyakunvat et al., 2016; Hua et al., 2019), the underlying mechanism remains largely unclear.

Recently, we reported viridicatumtoxins (VirA and VirB), a class of tetracycline-like antibiotics, strongly inhibit drug-resistant Gram-positive bacteria by directly binding to bacterial UPPS (Li et al., 2020). To further identify potential inhibitors of *E. faecalis* UPPS (*Efa*UPPS), we screened over 5,000 natural products by comparing their chemical structure properties, molecular weight, and hydrophobicity with our previously reported UPPS inhibitors VirA and VirB. Eventually, we found two natural compounds GA and NGA that display strong inhibition on *Efa*UPPS and have anti-*E. faecalis* activity.

In this study, we show that GA and NGA act as potential inhibitors toward several Gram-positive bacteria tested, such as *Enterococcus faecalis*, MRSA, and *Listeria monocytogenes*. The *in vitro* enzyme inhibition assays suggested that both of GA and NGA exhibited excellent inhibitory activity against *Efa*UPPS in a dose-dependent manner. Furthermore, based on the crystal structure of *Efa*UPPS and molecular docking studies revealed that GA and NGA can interact directly with the key residues of *Efa*UPPS binding pocket, which was further validated by site-directed mutagenesis and enzyme activity analyses. Importantly, in a mouse model of infection, GA and NGA can significantly reduce *E. faecalis*-induced inflammation. Collectively, we describe a key role for the natural products GA and NGA in fighting pathogenic bacteria through targeting UPPS, an essential enzyme involved in cell wall biosynthesis. Our findings provide new insights into the development and design of new antibiotics to combat drug-resistant bacterial infections through the prudent use of existing natural product resources.

## Materials and methods

### Strains, growth conditions, and chemical agents

*Enterococcus faecalis* ATCC29212 and *Staphylococcus aureus* ATCC29213 were purchased from the Fungal Genetics Stock Center. Methicillin-resistant *Staphylococcus aureus* (MRSA), *Listeria monocytogenes* ATCC19117*, Escherichia coli* ATCC25922, *Escherichia coli* BAS849, DH5α and BL21 were from Lin lab storage. The clinical strains used in this study were isolated and identified from Affiliated Hospital of Nanjing University of Chinese Medicine and Nanjing Drum Tower Hospital, China. All the bacteria strains were routinely cultured in Luria-Bertani (LB) broth media (1% tryptone, 0.5% yeast extract, and 1% NaCl) at 37°C, except as noted.

Two compound libraries (natural product library catalog#L1400 and FDA-approved drug library catalog#L1300) used for initial hit screen were purchased from Selleck Chemicals Co., Ltd. Gambogic acid (GA) and neogambogic acid (NGA) were purchased from Shanghai yuanye Bio-Technology Co., Ltd., China and were dissolved in DMSO.

### Protein expression, purification, and site-directed mutagenesis

*Enterococcus faecalis* undecaprenyl pyrophosphate synthase (*Efa*UPPS) recombination studies were performed as previously described (Li et al., 2020). Briefly, the full-length gene sequence of *Efa*UPPS was cloned into the *Nde*I and *Hind*III sites of pET28a vector under control of T7 promoter. The sequenced plasmid was then transformed into *E. coli* strain BL21(DE3) (Invitrogen) and the transformants were grown on LB broth media supplemented with 50 μg/mL kanamycin. A single colony of the transformant was then cultured in 100 mL of LB broth containing 50 μg/mL kanamycin overnight. The overnight cultures were inoculated into 1 L of LB broth containing 50 μg/mL kanamycin and continue to culture until reaching an OD_600_ of 0.8. Upon reaching the optimal growth states, the cultures were induced by addition of 1 mM isopropyl-β-D-thiogalactoside (IPTG), and then cultured at 16°C for 16 h. After centrifuging and collecting cells, the samples were subsequently re-suspended in buffer A (200 mM NaCl, 5% glycerol, 5 mM DTT, and 10 mM Tris–HCl, pH 7.9), and lysed using an AH-10013 cell disruptor (ATS). The lysate was separated by centrifugation (20,000 × g; 30 min at 4°C), and the supernatant was loaded onto a 5 mL column of Ni-NTA agarose (Qiagen) pre-equilibrated in buffer A. The column was washed 10 times with 5 mL buffer A containing 25 mM imidazole, and finally eluted with 50 mL buffer A containing 250 mM imidazole. Concentrated the eluted crude protein to ∼10 mg/mL by a 30 kDa MWCO Amicon Ultra-15 centrifugal ultrafilters (EMD Millipore), and purified by gel filtration chromatography on a HiLoad 16/60 Superdex 200 prep grade column (GE Healthcare). The yield of *Efa*UPPS was ∼5 mg/l, and purity was ∼95%, and stored in aliquots at −80°C.

The *Efa*UPPS site-directed mutagenesis was generated using QuikChange site-directed mutagenesis kit (Agilent) according to the manufacturer’s instructions. The purification of site-directed mutation was using a similar strategy as described above.

### Antibacterial activity

Antibacterial activities were performed by examining the minimal inhibitory concentrations (MICs), which was determined by a microtiter broth dilution method as previously described (Cotsonas King and Wu, 2009), with minor modifications. Briefly, 96-well plates (Corning) were inoculated with 100 μl of LB broth per well containing 7 × 10^5^ CFU/mL bacteria, followed by adding 100 μl of LB broth containing two-fold serial dilutions of the test compounds. Tetracycline (Tet) and ampicillin (Amp) and viridicatumtoxins were used as the positive controls. After 18 h of incubation, the MIC was determined to be the lowest concentration without visible bacterial growth. The results were the averages of at least three biological replicates.

### *Ex-vivo* growth inhibition assay

The *E. faecalis* ATCC29212 undecaprenyl pyrophosphate synthase genes, and the L91A and L146A mutation fragments were cloned into the *Bam*H I and *Hind* III digested pQE80Lvector, respectively. The resulting plasmids were transformed into *E. coli* BAS849, respectively. The strain transformed with the empty vector pQE80L was used as a negative control. A single colony of each transformant was inoculated into LB broth supplemented with 100 μg/mL ampicillin and incubated at 200 rpm, 37°C overnight. After diluted the cultures to 7 × 10^5^ CFU/mL by LB broth, 5 μl of each diluted cultures were dripped onto a LB agar plate supplemented with IPTG (0.5 mM), and GA or NGA (8 μg/mL) followed by cultivation at 37°C overnight, and then imaged. In the meantime, another 5 μl of each diluted cultures were diluted in 100 ul LB and coated on the relevant plates to check for bacterial CFUs.

### Bacterial activity test

The *E. faecalis* ATCC29212 were pre-cultured in LB broth at 200 rpm, 37°C overnight, and then the cultures were diluted to 7 × 10^5^ CFU/mL by LB broth. A 96-well microtiter plate was inoculated with 200 μl the diluted cultures per well containing 0.1 mg/mL Resazurin with or without 2 μg/mL GA or NGA, followed by incubation at 37°C for 24 h, and then imaged. Three biological replicates were performed for each sample. To visualized the potential bactericidal properties for GA and NGA, the 24 h of GA/NGA-pretreated *E. faecalis* were coated on the plate to continue cultured overnight. The bactericidal properties of the compounds were determined by counting the bacterial CFUs.

### UPPS inhibition assay

The UPPS inhibition assay was carried out by measuring the fluorescence of the reaction mixture in a black 96-well microtiter plate (Corning, United States). Briefly, the purified UPPS enzyme was mixed with 35 μM isopentenyl diphosphate (IPP), and 5 μM farnesyl diphosphate (FPP), as well as different concentrations of GA, NGA, or tetracycline (as a negative control) in 100 μL of reaction buffer containing 50 mM KCl, 0.5 mM MgCl_2_, 100 mM Tris–HCl (pH 7.5), 0.005% (w/v) Triton X-100, incubated for 30 min, at 37°C. After the indicated incubation period, the reaction was terminated by the addition of 10 μl of 0.5 M EDTA solution. Transferred 50 μl of the reaction mixture into a new 96-well microtiter plate, and quenched with an equal volume of Master Reaction Mix (Pyrophosphate Assay Kit, Sigma-Aldrich, USA) for 30 min. The UPPS inhibitory activity was performed by calculating the fluorescence percentage through determining the fluorescence absorbance of reaction mixture at 316 and 456 nm. Data of UPPS inhibitory activities were plotted versus compound concentrations on a semi-log scale using GraphPad Prism7. All the results were the means of at least three biological replicates.

The compound/substrate-UPPS binding competition experiment was performed as the above descriptions. The difference is that the reaction buffer containing 35 μM IPP, different concentrations of FPP ranged from 0–20 μM, and in the presence of 0/1/2/5 μM GA/NGA, respectively. Data were plotted with competitive inhibition model using GraphPad Prism7.

### Microscale thermophoresis binding assays

To characterize the binding affinity between *Efa*UPPS and GA or NGA, the MST binding assay was performed as previously described (Lin et al., 2020). In brief, His-tagged *Efa*UPPS and site-directed mutated *Efa*UPPS were labeled with the NT- 647-NHS dye using the Monolith NTTM Protein Labeling Kit RED-NHS (NanoTemper Technologies). Two-fold serially diluted GA or NGA was mixed with 10 nM of labeled *Efa*UPPS or its derivatives in 10 μl of binding buffer (137 mM NaCl, 2.7 mM KCl, 10 mM Na_2_HPO_4_, 1.8 mM KH_2_PO_4_, and 0.05% Tween-20, pH 7.8), incubated for 15 min, at room temperature. And then, loaded the mixtures into NT.115 premium coated capillaries (NanoTemper Technologies). MST trace signals were collected from the IR laser on to off for about 20 s, under the 5% LED power and high MST power mode. The change of molecular thermophoresis was monitored by standardized fluorescence value Fnorm, and the binding curve was plotted according to the values of Fnorm. The formed complex affinity was analyzed by MO Affinity Analysis software version 2.3. Apparent dissociation constants (K_d_) were calculated using nonlinear fitting assuming one specific binding site with the GraphPad Prism 7 software with the following formula: *Y =* B^∗^_Max_ X*/*KD + X.

(^∗^ where *B*_Max_ is the maximum theoretical specific binding; here *B*_Max_ = 1).

### Molecular docking study

The molecular docking study was carried out using Autodock4.2 package (Morris et al., 2009) as described in (Li et al., 2020) with slight modifications. Briefly, chain C of *Efa*UPPS crystal structure (Protein data bank accession number: 6LOI) was used as the rigid molecule. Autodock tool was used for arrangement of nonpolar hydrogens and partial atomic charges on the rigid molecule. The coordinates of GA and NGA were performed using CORINA Classic online service. A 70 × 90 × 90 grid box and 0.2 Å grid spacing centered roughly at the *Efa*UPPS substrate binding pocket was used as the searching space. To identify the protein-ligand interactions, 100 runs of Larmarckian Genetic Algorithm were performed. The results were clustered, ranked, and analyzed using PyMOL program.

### Animal experiments

All animal studies had been reviewed and approved by the Animal Ethical and Welfare Committee of Nanjing University of Chinese Medicine. 8-week-old male C57BL/6 J mice were housed in groups of four mice per cage in a 12/12-h day light cycle with free access to food and water under conditions of controlled humidity (50 ± 5%) and temperature (22 ± 2°C). Mice were adaptively raised for a week before experiments. To explore the effects of GA and NGA on *E. faecalis*, the mice were randomly divided into four groups including control, *E. faecalis*, *E. faecalis* + GA and *E. faecalis* + NGA. Mice received an oral dose (40 μM) of GA or NGA from 30 min after *E. faecalis* (1 × 10^10^ CFU) treatment for 19 days to sacrifice. The body weight of the mice was monitored daily. Afterwards mice spleens were collected and spleen weight was measured upon being sacrificed.

### Histochemistry assessment

Spleen tissue sections of mice were fixed in 4% paraformaldehyde, dehydrated in alcohol, embedded in paraffin and then sliced into thin 5 μm sections. The tissues were then stained with hematoxylin & eosin (H&E). The tissue images were observed under an inverted microscope (Vectra 3.0, PerkinElmer, United States).

### TNF-α production *in vivo*

Retro-orbital blood was collected from the mice in asepsis tubes before dissection. The blood was centrifuged at 3500 g for 20 min to extract serum, which was stored at −20°C awaiting subsequent analyses. TNF-α-levels in serum was measured by ELISA according to the instructions of the manufacturer (eBioscience, United States).

### Quantitative real-time polymerase chain reaction

Total RNA was extracted from the spleen of mice, and then the cDNA was synthesized with HiScript® II Q RT SuperMix for qPCR (Vazyme, China). qPCR was performed using the LightCycler®96 (Roche, Swiss). The qPCR primers of TNF-α (Forward: 5’-AAGCCTGTAGCCCACGTCGTA-3′ and Reverse: 5’-GGCACCACTAGTTGGTTGTCTTTG-3′) were designed and synthesized by GENERAY Biotechnology (Shanghai, China). GAPDH was used as the reference gene.

### Statistical analysis

All data were presented as mean ± standard error (SEM). Statistical analysis was shown as one-way ANOVAs with Tukey’s multiple comparisons by GraphPad Prism (GraphPad Software, USA). *P*<0.05 was considered as statistically significant.

## Results

### GA and NGA are strong inhibitors of *Efa*UPPS

**Figure 2:**
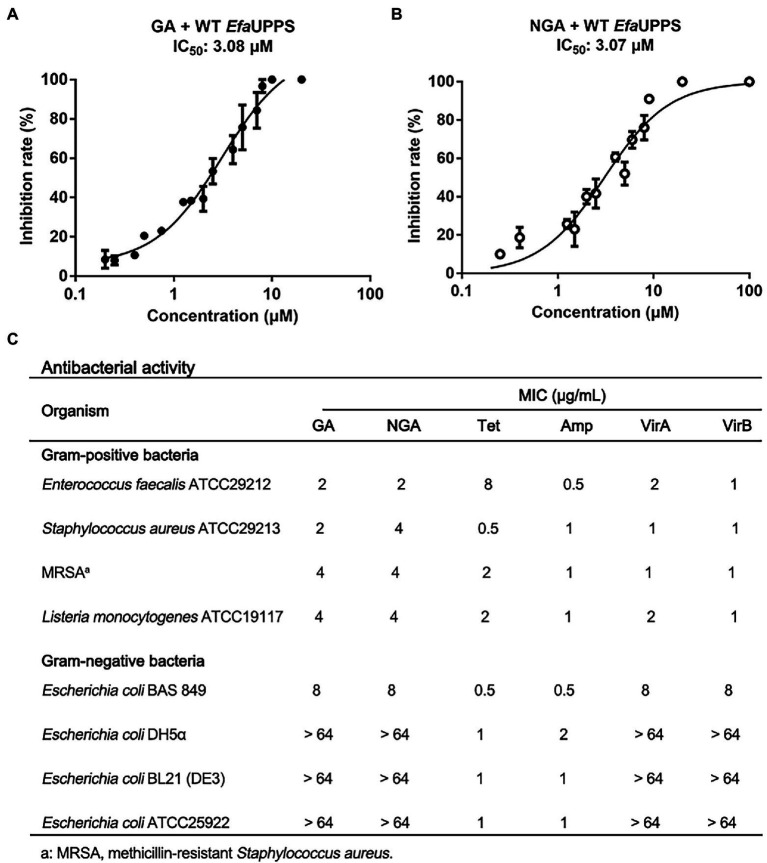
GA and NGA are strong inhibitors of *Efa*UPPS. **(A,B)** *In vitro* inhibitory activities of GA **(A)** and NGA **(B)** toward *Efa*UPPS. The calculated fluorescence percentages were plotted versus antibiotic concentrations on a semi-log scale (mean value ± SEM of three biological replicates). **(C)** Minimum inhibitory concentrations (MICs) of GA and NGA. Tetracycline (Tet), ampicillin (Amp) and viridicatumtoxins (VirA and VirB) were used as the positive controls. The data are presented as mean values from three independent biological replicates (*n* = 3, SD = 0).

To identify potential inhibitors of *E. faecalis* UPPS (*Efa*UPPS), two compound libraries containing over 5,000 natural products were *in silico* screened by comparing their chemical structure properties, molecular weight, and hydrophobicity with our previously reported UPPS inhibitors viridicatumtoxins A and B using a combination of AutoDock, Schrodinger, Biovia discovery studio, and virtual screening tools (Morris et al., 2009; Biovia, 2022; Sharma et al., 2022; Shivanika et al., 2022; Siva Kumar et al., 2022). One hundred twenty-seven candidate compounds were prioritized based on the property similarity and their inhibitory effects on the enzyme activity of the recombinant *Efa*UPPS (Supplementary Figure S1) were tested using an enzyme-coupled fluorescence assay. Intriguingly, among all the compounds tested, gambogic acid (GA) and neogambogic acid (NGA) inhibited *Efa*UPPS in a dose-dependent manner with the half maximal inhibitory concentration (IC_50_) values of 3.08 μM and 3.07 μM (Figures 2A,B), respectively, highly suggesting that GA and NGA target and inhibit *Efa*UPPS. To evaluate whether GA and NGA showed inhibitory effect on *E. faecalis*, we tested the antibacterial activities of GA and NGA by measuring their minimum inhibitory concentrations (MICs). As shown in Figure 2C, both GA and NGA exhibited promising antibacterial effects on *E. faecalis* with the MIC of 2 μg/mL in our broth microdilution assay. Taken together, both the enzyme inhibition assay and the growth inhibition experiment suggest that GA and NGA may exert their antibacterial effects through inhibiting UPPS activity, and that *Efa*UPPS is a potential target of GA and NGA.

To further verify that UPPS may be the potential target of GA and NGA *ex-vivo*, we transformed pQE80L-*Efa*UPPS^WT,^ pQE80L-*Efa*UPPS^L91A^, or pQE80L-*EfaUPPS*^L146A^ as well as the empty pQE80L vector as a control into *E. coli* BAS849, a strain with deficient outer-membrane structure, respectively. The results showed that the growth of *E. coli* transformed with the empty vector was completely inhibited by GA or NGA at 8 μg/mL, while overexpression of wild-type *Efa*UPPS antagonized the antibacterial effect and rescued the cell growth, conferring resistance on the cells. However, the growth of *E. coli* transformed with the pQE80L-*Efa*UPPS^L91A^ and pQE80L-*Efa*UPPS^L146A^ were showed moderate resistance to GA or NGA (Supplementary Figure S2). Collectively with the enzyme inhibition assay results of GA and NGA, these results provided evidence that GA and NGA may biologically target to *Efa*UPPS,

To determine an antibacterial dose baseline for GA and NGA on other pathogenic bacteria, the Gram-positive bacteria *Staphylococcus aureus*, methicillin-resistant *Staphylococcus aureus* (MRSA) and *Listeria monocytogenes*, as well as the Gram-negative bacteria *Escherichia coli* ATCC25922, BAS849 (a strain with deficient outer-membrane structure), DH5α and BL21 were assayed. According to the MIC results, both GA and NGA exhibited inhibitory activity toward *S. aureus* and *L. monocytogenes*, especially MRSA with the MIC values ranging from 2 μg/mL to 4 μg/mL. Furthermore, we also tested the MICs of the GA and NGA against 10 more of the clinical strains of *S. aureus*, *E. faecalis*, *L. monocytogenes,* respectively. These two compounds also showed high potency in growth inhibitory activity (Supplementary Figure S3). In addition, GA and NGA also showed moderate inhibitory activities against *E. coli* BAS849, a strain with deficient outer-membrane structure, with MICs at 8 μg/mL. However, GA and NGA had no inhibitory effect on Gram-negative bacteria *E. coli* at the highest concentration tested (64 μg/mL; Figure 2C). Furthermore, to test the potential bactericidal properties for GA and NGA, we coated the 24 h GA/NGA-pretreated *E. faecalis* cultures on the plate to continue cultured for overnight. As a result, the cultures without GA/NGA treatment were full of plates, and only a few bacteria settled on the plate after GA/NGA treatment, which was far less than the initial inoculations (7 × 10^5^ CFU/mL), indicating that GA and NGA have potential bactericidal effects at 2 μg/mL (Supplementary Figure S4). These results demonstrated that GA and NGA are strong inhibitors of Gram-positive bacteria.

**Figure 3:**
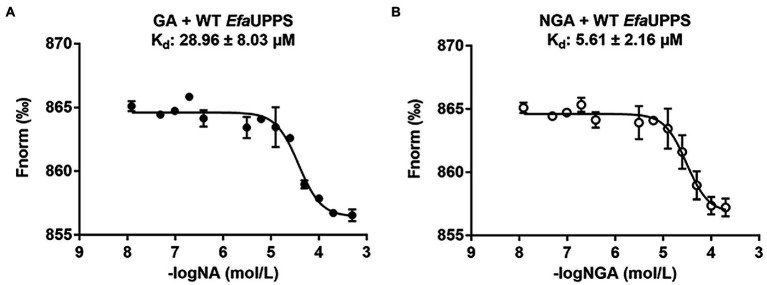
MST analyses of the binding of GA **(A)** and NGA **(B)** to *Efa*UPPS. The titration of GA or NGA ranged from 3.05 nM to 200 μM with a constant concentration of the *Efa*UPPS at 5 nM. The results of GA were plotted with solid dots, while the results of NGA were plotted with hollow dots. The data are presented as means of three independent biological replicates. Error bars represent mean ± SEM of *n* = 3 experiments.

To determine whether the *Efa*UPPS enzyme is inhibited by direct binding of GA or NGA, microscale thermophoresis (MST) analysis was performed. The titration of *Efa*UPPS with GA followed an endothermal heat change profile, giving rise to a sigmoidal binding curve. The estimated dissociation constant K_d_ of *Efa*UPPS with GA using nonlinear fitting binding model was at micro-molar range (∼28.96 μM; Figure 3A), indicating a moderate affinity between the ligand GA and *Efa*UPPS. The binding between NGA and *Efa*UPPS was also analyzed by MST. Consistent with the results for GA, the equilibrium dissociation coefficient for NGA and *Efa*UPPS was determined to be K_d_ = 5.61 ± 2.16 μM (Figure 3B), which is about 5-fold lower than that for GA, suggesting that both GA and NGA inhibit *Efa*UPPS activity through direct binding, and the affinity of NGA is higher than that of GA. Therefore, understanding the difference in molecular interaction of GA/NGA with *Efa*UPPS would help to explain the obvious difference in binding affinity for *Efa*UPPS.

### Molecular docking of GA and NGA with *Efa*UPPS

**Figure 4:**
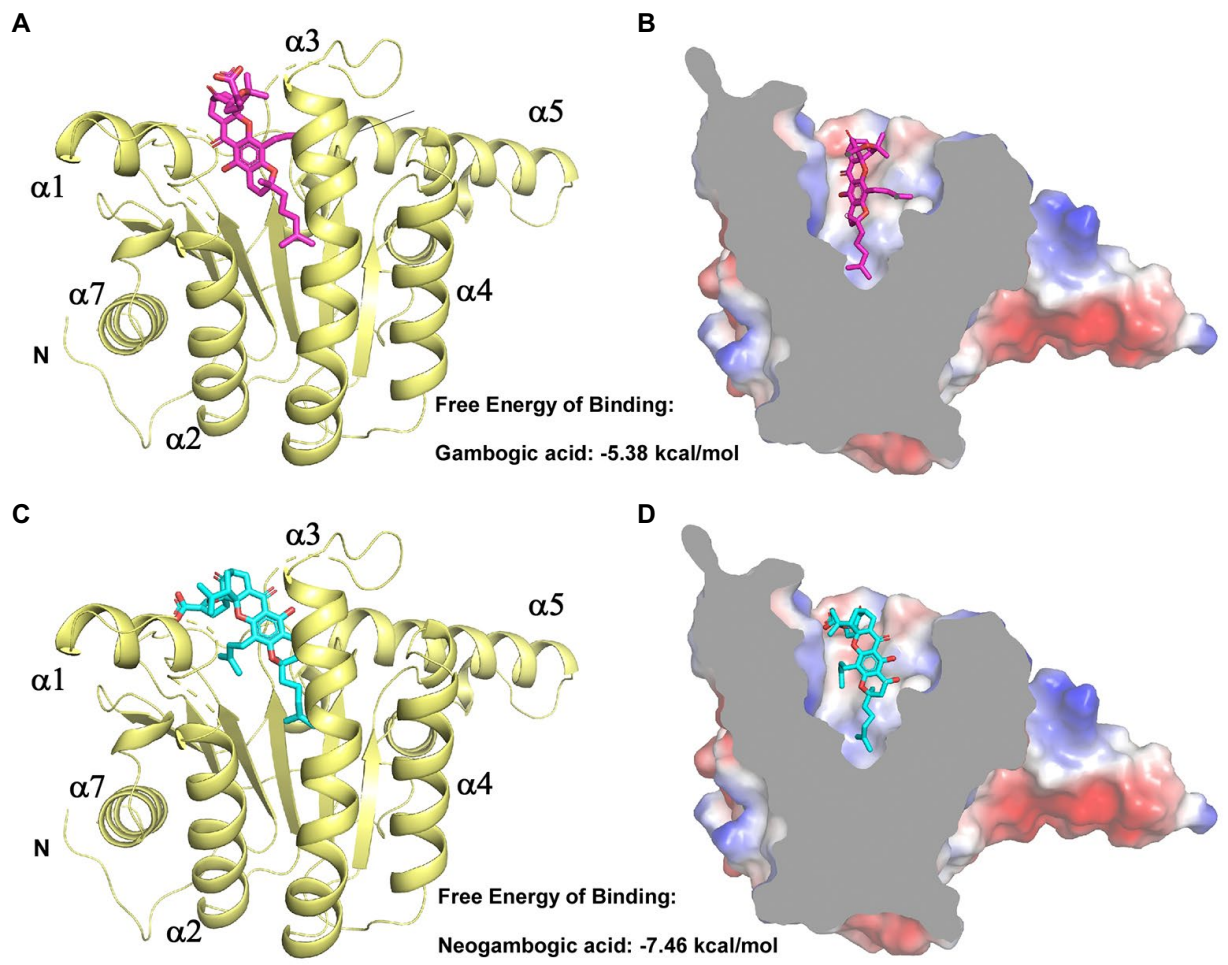
Molecular docking of GA and NGA with *Efa*UPPS. **(A,C)** Energetically favorable docking models of GA or NGA to *Efa*UPPS (PDB ID: 6LOI): purple, GA carbon atoms; cyan, NGA carbon atoms; red, oxygen atoms; blue, nitrogen atoms. **(B,D)** Surface electrostatic potential of *Efa*UPPS in complex with GA or NGA. Colors of compound models are the same as those in part **(A,C)**.

We next aimed to experimentally verify and explore the interactions between GA/NGA and *Efa*UPPS by analyzing the co-crystallization data. However, our trials to obtain co-crystals were unsuccessful possibly due to the poor aqueous solubility of GA/NGA and improper crystal packing. Alternatively, we performed a computer-aided molecular docking approach to evaluate the potential interactions between *Efa*UPPS and GA/NGA. For the *Efa*UPPS enzyme, the overall structure comprises of seven surrounding α-helices and a central β-sheet with six parallel strands (Figures 4A,C), while for GA and NGA, the chemical structures of them are similar. The polyprenylation of xanthone skeleton allows the compounds to possess both hydrophilic and hydrophobic moieties. The hydrophobic face is dominated by the alkenyl chains and a segment of the polycyclic ring system. The hydrophilic face includes several carboxylate ions and oxygen atoms. The molecular docking studies (Figure 4) revealed that both GA and NGA can occupy the substrate FPP binding pocket of *Efa*UPPS, with an estimated free binding energy of −5.38 kcal/mol for GA and − 7.46 kcal/mol for NGA, respectively. The hydrophobic xanthone rings of GA and NGA are positioned in the active site of *Efa*UPPS (Figures 4B,D), which may have potential direct hydrogen-bonding interactions with the surrounding hydrophilic amino acid residues in *Efa*UPPS.

### Key amino acid residues of GA and NGA binding pocket in *Efa*UPPS

**Figure 5:**
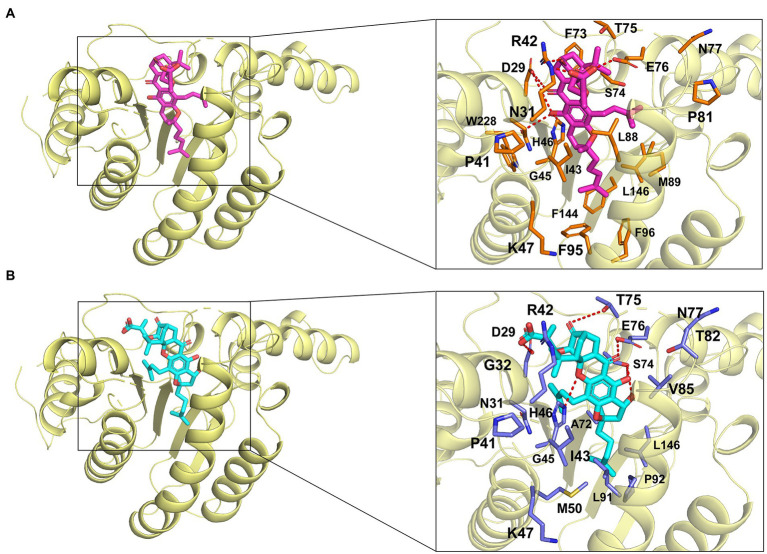
Simulated molecular interactions between GA/NGA and *Efa*UPPS. **(A,B)** Overall structure of *Efa*UPPS enzyme in complex with GA (purple, **A**) or NGA (cyan, **B**). The close-up views of simulated GA/NGA-*Efa*UPPS interactions in the binding pocket are highlighted in black boxes: red stick, oxygen atom; blue stick, nitrogen atom.

In the molecular docking model, GA and NGA are surrounded by hydrophilic residues D29, N31, R42, H46, K47, S74, T75, E76, and N77, together with hydrophobic residues P41, I43, G45, and L146 of *Efa*UPPS (Figures 5A,B). Furthermore, the docking model revealed that the unique lipophilic spirocyclic xanthone moiety of GA and NGA interacts with the residues D29, N31, R42, H46, K47, S74, T75, E76, and N77, forming a hydrophobic cleft (Figure 5). For *Efa*UPPS-GA complex, potential hydrogen bonds interactions are probably formed between D29 and the carbonyl group on ring 2 and the hydroxyl group on ring 3, between N31/H46 and the hydroxyl group on ring 3, between R42 and the carbonyl group on ring 1, and between E76 and the carboxyl group on the side chain connected to ring 1 (Figure 5A). Despite the similarities between the chemical structures of GA and NGA, the hydrogen bonds formed between *Efa*UPPS and NGA appear to be different from those in *Efa*UPPS-GA, which may explain the differences in the binding affinity of *Efa*UPPS with GA or NGA. For example, the residue D29 forms a hydrogen bond with the carboxyl group on the side chain of ring 1 in NGA instead of the carbonyl group on ring 2 in GA. Other hydrogen bonds in the *Efa*UPPS-GA complex are established between H46 and the ether group on ring 2, between S74 and the hydroxyl groups on ring 3 and ring 4, between T75 and the carbonyl on ring 1, and between E76 and the carbonyl group on ring 2 (Figure 5B). Collectively, the above results demonstrated that GA and NGA partially occupied the same binding pocket as UPP synthase’s natural substrate, FPP. To this end, enzyme assays confirmed that GA and NGA compete with FPP for binding to the *Efa*UPPS enzyme (Supplementary Figure S5), thus preventing the biosynthesis of UPP.

**Figure 6:**
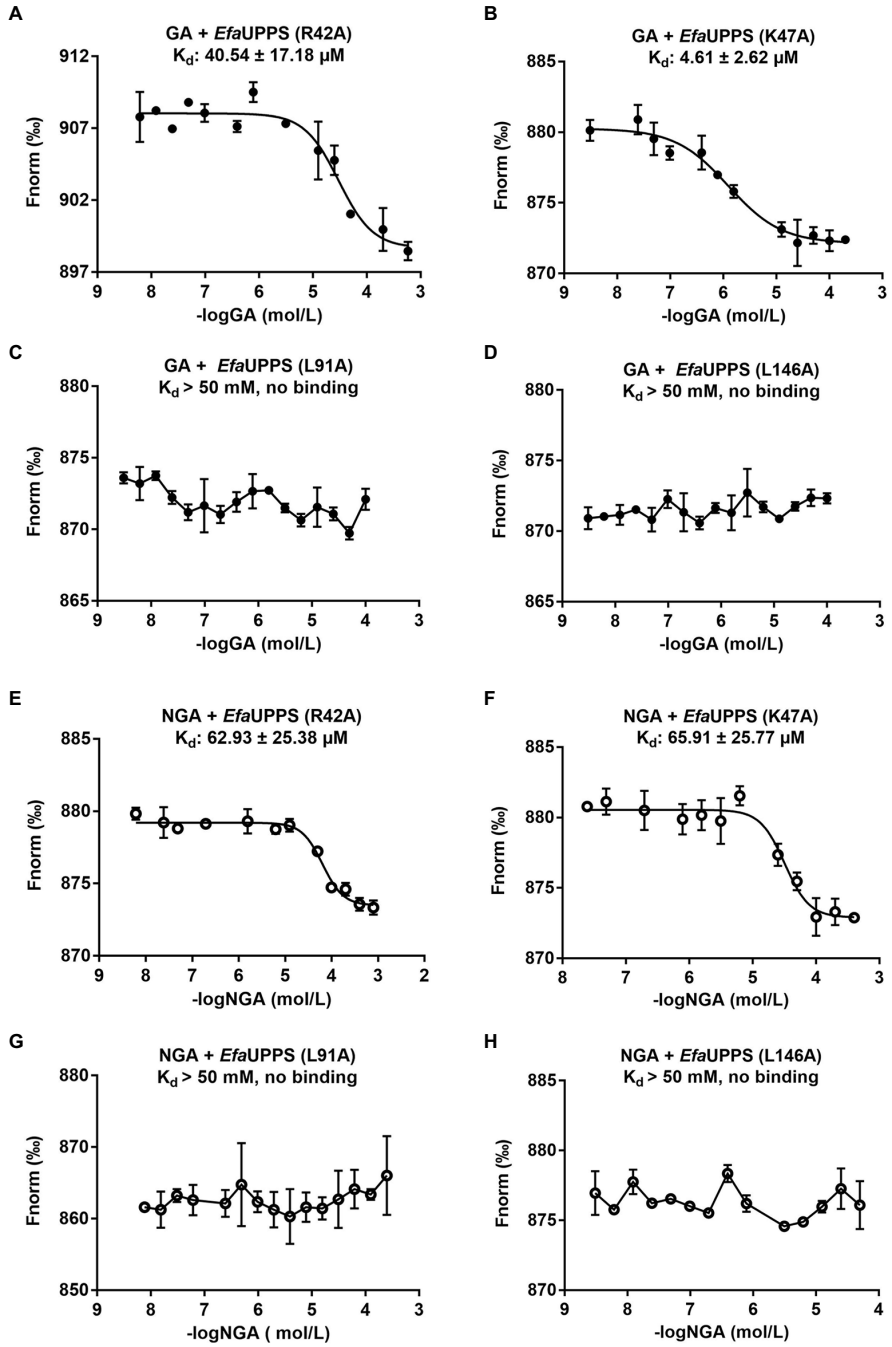
MST analyses of the binding of GA/NGA to wild-type and mutated *Efa*UPPS proteins (**A–H**). The concentration of wild-type *Efa*UPPS and its indicated mutants is kept constantly at 5 nM, while the GA/NGA concentration ranged from 3.05 nM to 200 μM. The results of GA were plotted with solid dots, while the results of NGA were plotted with hollow dots. The data are presented as means of three independent biological replicates. Error bars represent mean ± SEM of *n* = 3 experiments.

To determine whether the critical amino acid residues identified in the molecular docking model are required for the interactions between GA/NGA and *Efa*UPPS, we created 15 single mutations for the residues in the *Efa*UPPS binding pocket, and the effects of the mutations on *Efa*UPPS binding affinity and enzymatic activity were evaluated. As shown in Supplementary Figure S6, three *Efa*UPPS mutants (I43A, L91A, and L146A) significantly decreased the inhibitory effect of GA on *Efa*UPPS enzyme activity. Furthermore, we measured the IC_50_ values of GA and NGA on the activity of other *Efa*UPPS mutants (R42A, K47A, L91A, L146A, and I43A). The reduced *Efa*UPPS enzyme activity of K47A mutant (Supplementary Figure S7) may be responsible for the increased IC_50_ values of K47A mutant in the presence of GA. The IC_50_ values of GA on the activities of the three mutants (I43A, L91A, and L146A) were ∼ 30 μM (Supplementary Figures S8A–D, S9A), which is about 10-fold higher than that of the wild-type (∼3 μM) (Figure 2A). In agreement with these results, there was no detectable binding affinity (K_d_ > 50 mM, no binding) of GA to I43A, L91A and L146A mutants in the MST binding analysis (Figures 6C,D; Supplementary Figure S9B), demonstrating that Ile43, Leu91 and Leu146 are the key amino acid residues for the interaction between *Efa*UPPS and GA, and the hydrophobic isoleucine and leucine residues promote the formation of stable hydrophobic interactions between *Efa*UPPS and GA. The R42A mutation does not have significant impact on *Efa*UPPS activity (IC_50_ = 5.39 μM for R42A *vs* IC_50_ = 3.08 μM for the wild-type *Efa*UPPS) as revealed by its binding affinity (K_d_ = 40.54 ± 17.18 μM for R42A *vs* K_d_ = 28.96 ± 8.03 μM for the wild-type *Efa*UPPS) (Figures 3A, 6A). Unexpectedly, we observed that the K47A mutation promoted the binding affinity of GA to *Efa*UPPS (K_d_ = 4.61 ± 2.62 μM) (Figure 6B), which is about 6-fold lower than that of the wild-type (K_d_ = 28.96 ± 8.03 μM) (Figure 3A), however, it seems to have no obvious difference on the inhibitory effect of enzyme activity compared with the wild type enzyme (Supplementary Figure S6), possibly owing to that the *Efa*UPPS are fully saturated by GA molecules.

Like GA, the *Efa*UPPS mutants R42A, K47A, L91A, and L146A significantly reduced the inhibitory effect of NGA on *Efa*UPPS (Supplementary Figure S10). Similarly, the mutants R42A, K47A, L91A, and L146A were selected to determine the binding affinity of NGA to the *Efa*UPPS mutants. In accordance with the enzyme activity results, the equilibrium dissociation coefficient (K_d_) of NGA to the *Efa*UPPS R42A and K47A mutants were determined to be 62.93 ± 25.38 μM and 65.91 ± 25.77 μM, respectively (Figures 6E,F), which are about 10-fold higher than that of the wild-type *Efa*UPPS (K_d_ = 5.61 ± 2.16 μM) (Figure 2B). The results suggested that both R42 and K47 residues contribute to the interaction between NGA and *Efa*UPPS. Most importantly, the *Efa*UPPS mutants L91A and L146A blocked the binding of NGA to *Efa*UPPS (K_d_ > 50 mM, no binding) (Figures 6G,H). The IC_50_ values of NGA toward L91A and L146A mutants were also about 30 μM (Supplementary Figures S9G,H), which is about 10-fold higher than that of the wild-type (∼3 μM) (Figure 3B). Overall, these observations confirmed that the residues L91 and L146 in *Efa*UPPS are essential to stabilize the interactions between GA/NGA and *Efa*UPPS. In conclusion, the site-directed mutagenesis analysis validated the key active sites of *Efa*UPPS for GA/NGA binding and suggested that changes in the hydrophobicity of xanthone moiety may enhance the interactions between GA/NGA and UPPS, thus increasing its antibacterial ability.

### Effects of GA and NGA on the *Enterococcus faecalis* infected mouse model

**Figure 7:**
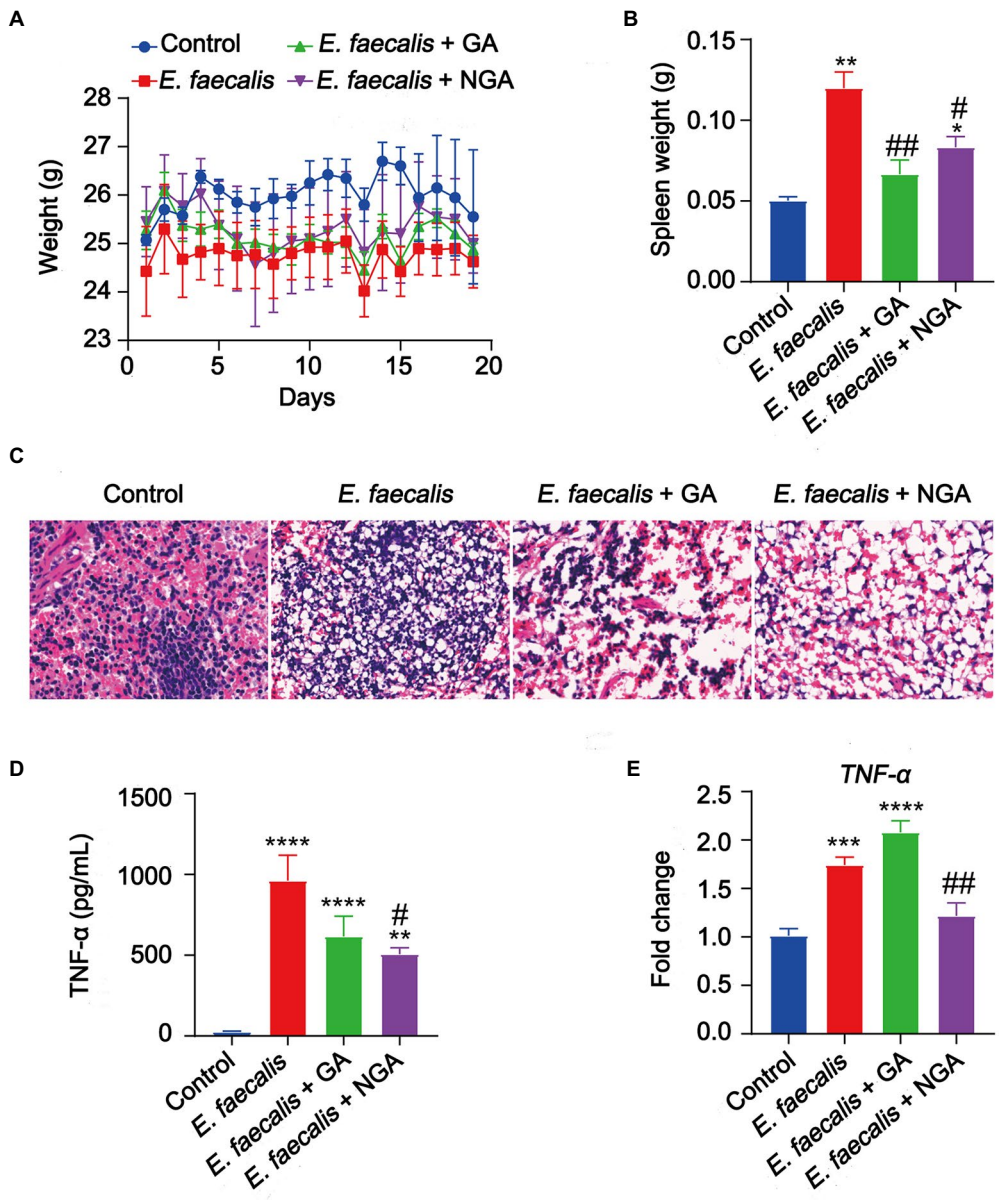
Supplementation with GA or NGA improved inflammation induced by *E. faecalis*. **(A)** Body weight changes of mice in different groups. **(B)** Spleen weight were analyzed. **(C)** Representative histological images of spleen tissues by H&E staining. Amplification, 10 × 40. **(D)** The serum concentration of TNF-α was detected. **(E)** The mRNA expression of TNF-α in spleen. The data are presented as the mean ± SEM and were analyzed by ordinary one-way ANOVA with Tukey’s multiple comparisons. *p < 0.05, **p < 0.01, ***p < 0.001, ****p < 0.0001, compared with control; #*p <* 0.05, ##*p <* 0.01, compared with *E. faecalis* group.

To evaluate the antibacterial activity of GA and NGA *in vivo*, a mouse model infected with *E. faecalis* was applied. The *in vivo* assays showed that oral intake of GA or NGA had no observable effect on body weight of *E. faecalis* infection mice (Figure 7A). However, both GA and NGA significantly relieved splenomegaly (Figure 7B). Furthermore, *E. faecalis* is observed to cause substantial spleen inflammation in the mouse infection model. In contrast, spleen slides from GA or NGA administration revealed reduced signs of inflammation (Figure 7C). To investigate the effect of GA/NGA on inflammatory cytokine production, TNF-α-levels in serum was measured by ELISA. Compared to the control group, TNF-α-levels in serum was increased in *E. faecalis* infection mice, while NGA supplementation significantly reduced the TNF-α-levels in serum (Figure 7D). In agreement with our ELISA results, qPCR analyses of spleen from *E. faecalis* infection mice after NGA treatment revealed significant downregulation of TNF-α (Figure 7E). These findings demonstrated that GA and NGA can significantly reduce *E. faecalis*-induced inflammation in the mouse model, with NGA being more effective than GA in reducing TNF-α production.

## Discussion

Cell wall is essential for the maintenance of cell shape and structural integrity of bacterial cells. Given that their absence from human hosts and their key role in bacterial viability, cell walls are ideal targets for antibacterial development (Schneider and Sahl, 2010). In the present study, we characterized the antibacterial activities and the potential mechanism of action of two gamboge-derived bioactive compounds GA and NGA, which exerts antibacterial effect through targeting undecaprenyl diphosphate synthase (UPPS) involved in bacterial cell wall biosynthesis. Using microscale thermophoresis, structure-based molecular docking studies in combination with enzyme activity assays, we found that both GA and NGA could occupy the substrate binding pocket of *Efa*UPPS, and that both showed micro-molar level of affinity with *Efa*UPPS. Most importantly, GA and NGA not only exhibited antibacterial *in vitro*, but also improved *E. faecalis*-induced inflammation in a mouse infection model. Our findings provide a solid foundation for structural optimization of GA/NGA in order to develop efficient antibacterial agents and improve treatment success rates in clinical practice.

It is well-known that natural products play a critical role in drug discovery and development (Newman and Cragg, 2020). An increasing amount of evidence has indicated that natural bioactive compounds GA and NGA have a variety of biological activities, such as anticancer, antioxidant, anti-inflammatory, and anti-infectious activities (Li et al., 2010; Yu et al., 2012; Chi et al., 2013; Yang and Chen, 2013; Chaiyakunvat et al., 2016; Pandey et al., 2016; Zhang et al., 2016; Hatami et al., 2020; Liu et al., 2020; Zhou et al., 2020). Unlike other natural anticancer compounds, GA has proved to be effective at the nanomolar level (Zhang et al., 2004; Luo et al., 2019). Our present study confirmed that GA and NGA have effective antibacterial effects toward *E. faecalis* through the inhibition of *Efa*UPPS at the micro-molar levels. Although the present data showed that the binding affinity of NGA to UPPS is slightly higher than that of GA, the difference in binding affinities may be caused by a potential hydrogen bond interaction between the hydroxyl group located at ring 4 of NGA and residue Ser74 of *Efa*UPPS based on the docking results (Figure 4). This interaction may enhance and stabilize the binding of NGA to the *Efa*UPP, while no such interaction for GA. To some extent, we speculated that the substrate binding pocket of UPPS may have been saturated by GA or NGA. Even though their affinities with UPPS are somewhat different, this will not change their strong inhibition of UPPS activity. Based on the satisfied safety profile and large therapeutic index of GA (Chi et al., 2013) as well as its general pharmacological effects (Zhao et al., 2010), various chemical modifications have been performed to make GA as a better antitumor agent (Zhang et al., 2004) previously. Our study here provides a theoretical basis for the development of GA and NGA into promising antibacterial agents.

It is worth noting that although GA and NGA have been reported to show anti-MRSA activity *in vitro*, their biological target remains largely unknown (Chaiyakunvat et al., 2016; Hua et al., 2019). MRSA poses a significant and enduring problem to the medical field all over the world, which has already attracted the attention of many researchers worldwide. Hua and colleagues showed that GA and NGA can reduce the expression levels of *S. aureus* virulence factors by inhibiting the *saeRS* two-component, thus achieving inhibition of MRSA (Hua et al., 2019). Different from their findings, we found that GA and NGA exhibited excellent inhibitory activity toward Gram-positive bacteria *E. faecalis* by targeting UPPS, which may also be an important target for the inhibition of MRSA. The *S. aureus sae* locus contains a classical two-component signaling module (TCS), of which SaeS is a receptor kinase and SaeR is a response regulator (Giraudo et al., 1999). We found two proteins in *E. faecalis* ATCC29212 (the NCBI sequence ID: EJS8068.91and OOC91500.1) showed 31 and 28% identities with *S. aureus* SaeR and SaeS, respectively. The EJS8068.91 is a predicted DNA-binding response regulator, while OOC91500.1 encodes a two-component sensor histidine kinase in *E. faecalis*, which play potential roles similar to that of SaeR and SaeS in *S. aureus*. Whether the antibacterial effect of GA and NGA are related to the proteins requires further study. Moreover, it is reported that GA and NGA can effectively inhibit septicemic in a mouse sepsis model caused by *S. aureus*. However, GA and NGA previously failed to improve inflammation in the heart, spleen and kidneys of MRSA ATCC 33951-infected mice (Hua et al., 2019). In the current study, we found that the production of proinflammatory cytokine, TNF-α, was induced obviously in *E. faecalis* infection mice, while the underlying mechanism needs further investigation. TNF-α is a marker of M1 macrophage, which elicits rapid proinflammatory responses to infection and tissue damages (Yoon et al., 2017; Pan et al., 2022). TNF release is typically activated by a variety of chemicals, the most active of which is bacterial LPS endotoxin (Wang et al., 2023). Our study here revealed that GA and NGA can significantly improve *E. faecalis*-induced undesirable inflammation. The treatment of GA and NGA can inhibit the expression of proinflammatory cytokines, especially TNF-α mainly produced by macrophages. Therefore, we considered that GA and NGA may reduce the inflammation through directly targeting on macrophages. In conclusion, the *in vivo* and *in vitro* experiments revealed that GA and NGA are excellent antibacterial lead compounds that can effectively target bacterial UPPS.

In recent years, UPPS has become an attractive target for antibacterial compound screening. At present, a few compounds that bind to different bacterial UPPSs have been designed and chemically synthesized, including bisphosphonates, tetramic acids, diketoacids, and benzoic acids (Guo et al., 2007; Kuo et al., 2008; Peukert et al., 2008; Durrant et al., 2011; Zhu et al., 2013), while the natural UPPS inhibitors are rarely reported. These synthesized UPPS inhibitors usually have issues in safety and pharmacological side effects even after multiple rounds of structural optimization, and thus none of them is developed into approved antibiotics. In contrast, commercial drugs and lead compounds that passed phase I clinical trials have less such issues. Therefore, discovering new pharmacological functions of old drugs (drug repurposing) is a very economical and effective strategy for the “ready-to-use drugs” (Nunes-Alves, 2015; Beijersbergen, 2020). Based on this theory, to find novel potential antibacterial compounds, this study screened the natural product and FDA-approved drug libraries and characterized the detailed drug-enzyme interactions mechanism based on rigid docking with the *Efa*UPPS crystal structure. The docking results clearly showed that it was the most possible for GA or NGA to bind within the substrate pocket (92 and 90% predicted binding conformations located within substrate pocket, data not shown) probably since only the substrate binding pocket can accommodate such a large size and chemical properties as GA/NGA and lower binding free energies can be obtained. Importantly, GA and NGA are bioactive compounds isolated from gamboge, which has a long history of use as traditional Chinese medicines (Deng et al., 2012; Pandey et al., 2016; Hatami et al., 2020). More importantly, GA has been certified by the China Food and Drug Administration for clinical trials (Chi et al., 2013; Yang and Chen, 2013). Therefore, the combination of GA or NGA with traditional antibacterial agents may be an effective strategy for the treatment of bacterial infections such as MRSA and VRE.

The emergence of antibiotic resistance has reduced the effectiveness of existing antibiotic drugs and posed a great threat to global public health. As the discovery rate of new antibiotics has decreased significantly, repurposing the approved drugs is becoming an alternative to new antibiotics (Nunes-Alves, 2015; Beijersbergen, 2020). Moreover, rational optimization and remodeling of existing antibiotic scaffolds is another option. Herein we reported the biological targets and mechanism of action of the natural products GA and NGA isolated from gamboge. Our studies confirmed that GA and NGA strongly inhibit bacterial growth by interacting with the key amino acid residues and occupying the substrate binding pocket of UPPS. This research will serve as inspiration for rational optimization or reuse of natural GA/NGA scaffolds in the development and design of new antibiotics.

## Data availability statement

The original contributions presented in the study are included in the article/Supplementary material, further inquiries can be directed to the corresponding authors.

## Ethics statement

The animal study was reviewed and approved by the Ethics Committee of Nanjing University of Chinese Medicine.

## Author contributions

YC, ZS, and WL designed experiments, analyzed the data, and wrote the manuscript. ML, LW, CL, PC, YJ, JL, and FG performed the bulk of the experiments and contributed to protein expression, purification, and crystallization. WL contributed to molecular docking assay and conceived the project. All authors contributed to the article and approved the submitted version.

## Funding

This work was funded by the National Natural Science Foundation of China (82072240, 81903756, and 32270192), Jiangsu Province of China (BK20190798 to WL), the Open Project of State Key Laboratory of Drug Research, Shanghai Institute of Materia Medica, Chinese Academy of Sciences (No. SIMM2205KF to WL), the Open Project of State Key Laboratory of Microbial Resources, Institute of Microbiology, Chinese Academy of Sciences (No. SKLMR-20220704 to WL), the Open Project of Chinese Materia Medica First-Class Discipline of Nanjing University of Chinese Medicine (No. 2020YLXK008 to WL), the Fok Ying Tung Education Foundation, and Jiangsu Specially-Appointed Professor Talent Program to WL.

## Conflict of interest

The authors declare that the research was conducted in the absence of any commercial or financial relationships that could be construed as a potential conflict of interest.

## Publisher’s note

All claims expressed in this article are solely those of the authors and do not necessarily represent those of their affiliated organizations, or those of the publisher, the editors and the reviewers. Any product that may be evaluated in this article, or claim that may be made by its manufacturer, is not guaranteed or endorsed by the publisher.
