# Supplementary material for: Investigations into the antibacterial effects and potential mechanism of gambogic acid and neogambogic acid
Source: Front Microbiol. 2022 Dec 12;13:1045291. doi: 10.3389/fmicb.2022.1045291 (PMC9791066; doi:10.3389/fmicb.2022.1045291)
Supplement: Supplementary file 1 [file Data_Sheet_1.docx]

**Supplementary Materials**

**Investigations into the Antibacterial effects and Potential Mechanism of Gambogic Acid and Neogambogic Acid**

**Mingzhu Li^1,2,#^, Yuan Chen^2,#,*^, Lijuan Wang^2^, Chujie Lu^1,3^, Peiying Chen^2^, Yuanling Jin^2^, Jiacong, Li^2^, Fei Gao^2^, Zhuo Shang^6,*^, Wei Lin^1,2,5,6,*^**

^1^Jiangsu Collaborative Innovation Center of Chinese Medicinal Resources Industrialization, Nanjing 210023, China

^2^ Department of Pathogen Biology, School of Medicine & Holistic Integrative Medicine, Nanjing University of Chinese Medicine, Nanjing 210023, China.

^3^ School of Pharmacy, Nanjing University of Chinese Medicine, Nanjing 210023, China

^4^ School of Pharmaeutical Sciences, Shandong University, Jinan 250012, China

^5^ State Key Laboratory of Drug Research, Shanghai Institute of Materia Medica, Chinese Academy of Sciences, Nanjing 210023, China

^6^ State Key Laboratory of Microbial Resources, Institute of Microbiology, Chinese Academy of Sciences, Nanjing 210023, China

#, Equal contribution.

***, Correspondence:**

Corresponding Authors

[weilin@njucm.edu.cn](mailto:weilin@njucm.edu.cn) OR [zshang@sdu.edu.cn](mailto:zshang@sdu.edu.cn) OR yuanchen@njucm.edu.cn

**
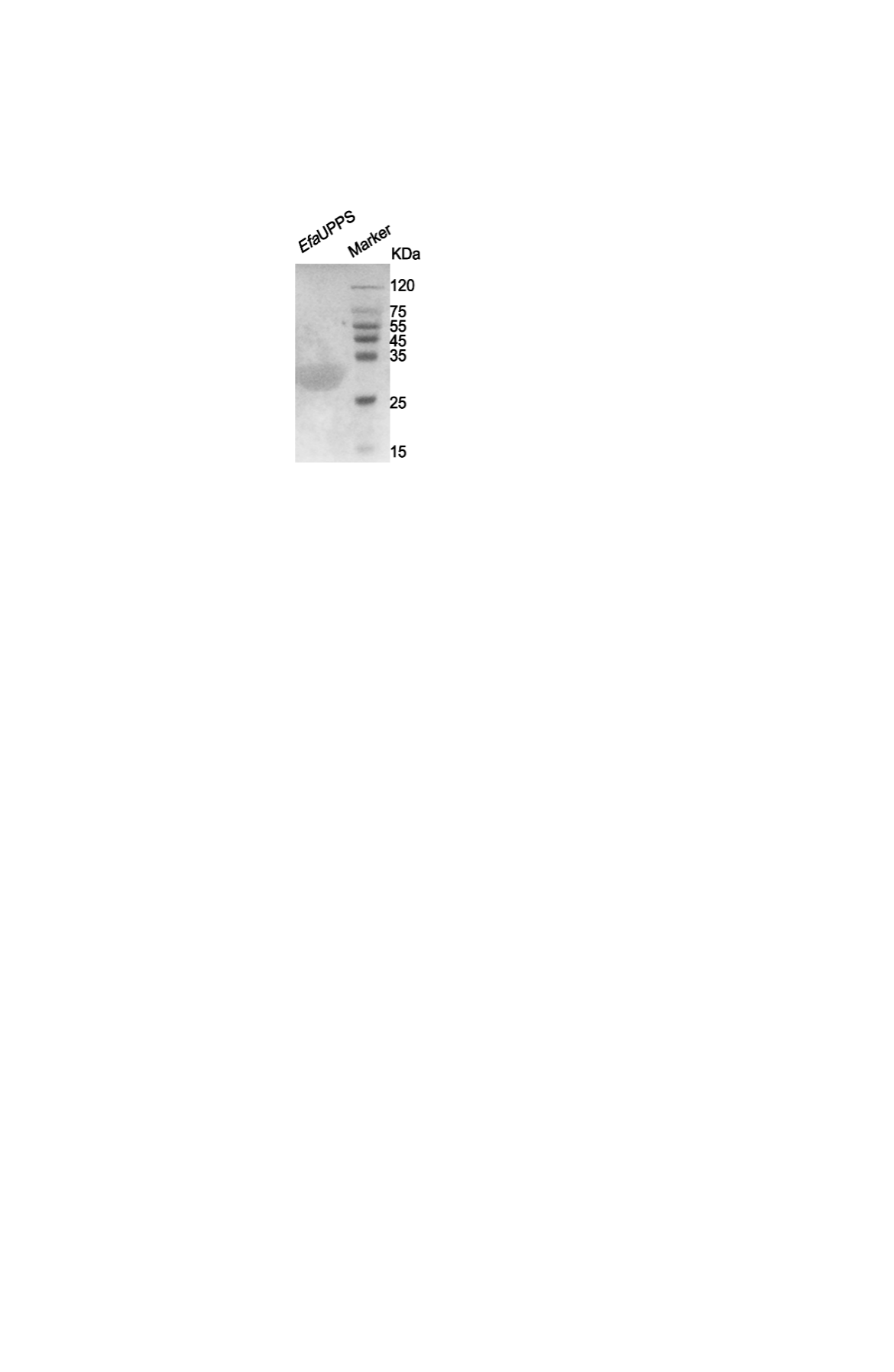
**

**Supplementary Figure S1.** Sodium dodecyl sulfate polyacrylamide gel electrophoresis (SDS-PAGE) of *Efa*UPPS protein (~30 kDa) overexpressed in *E. coli* BL21(DE3).


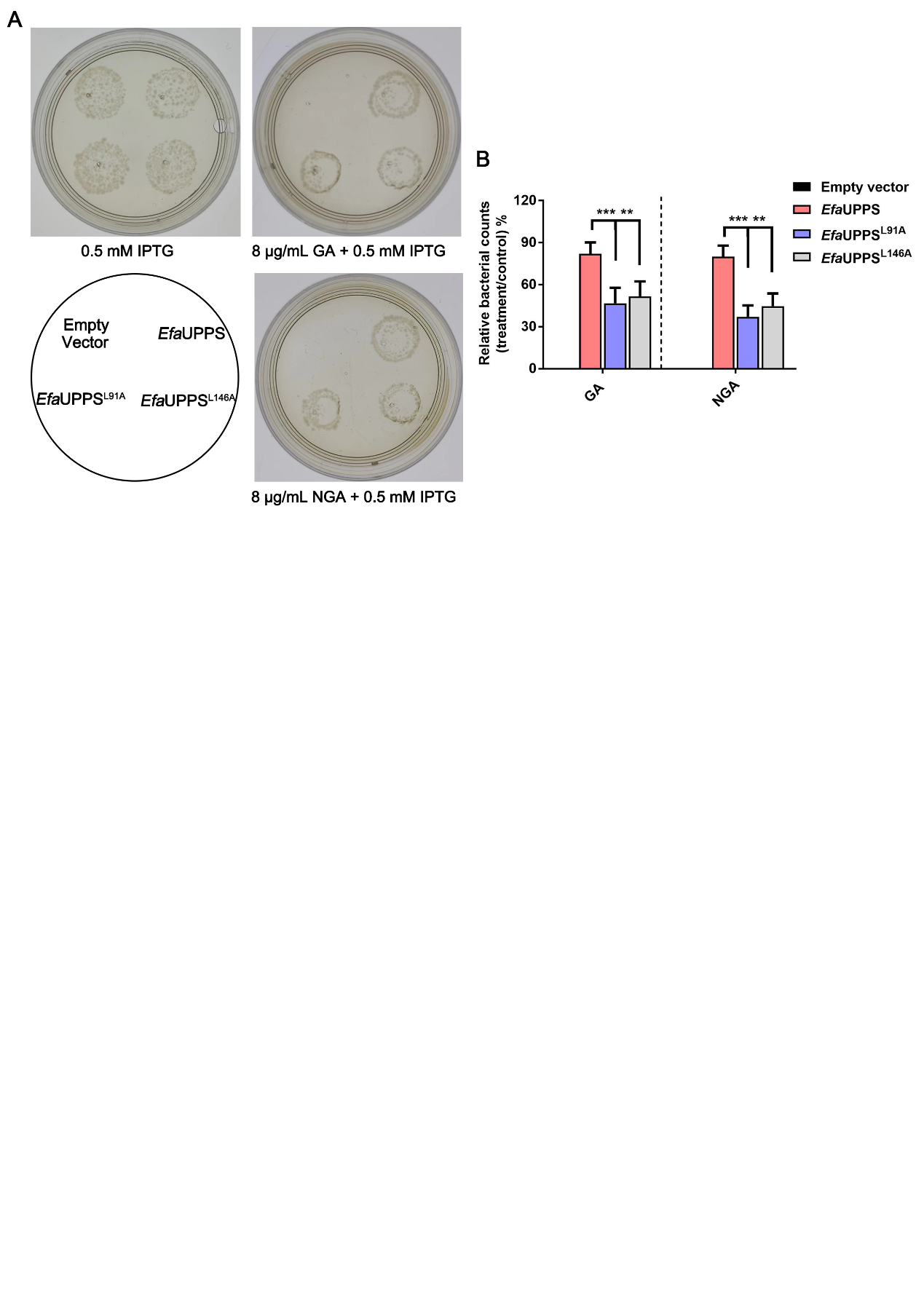


**Supplementary Figure S2. (A)** *Ex-vivo* growth inhibition assays of GA and NGA against *E. coli* BAS849 transformed with wild-type *Efa*UPPS, *Efa*UPPS^L91A^, and *Efa*UPPS^L146A^. The strain transformed with the empty vector pQE80L was used as the negative control. **(B)** Quantification of the relative bacterial counts for panel **A**, the data are presented as the percentage of total bacterial counts after GA/NGA treatment *vs* control (IPTG only). (**, *P*<0.01; ***, *P*<0.001). The data are presented as the mean ± SEM of three independent biological replicates.


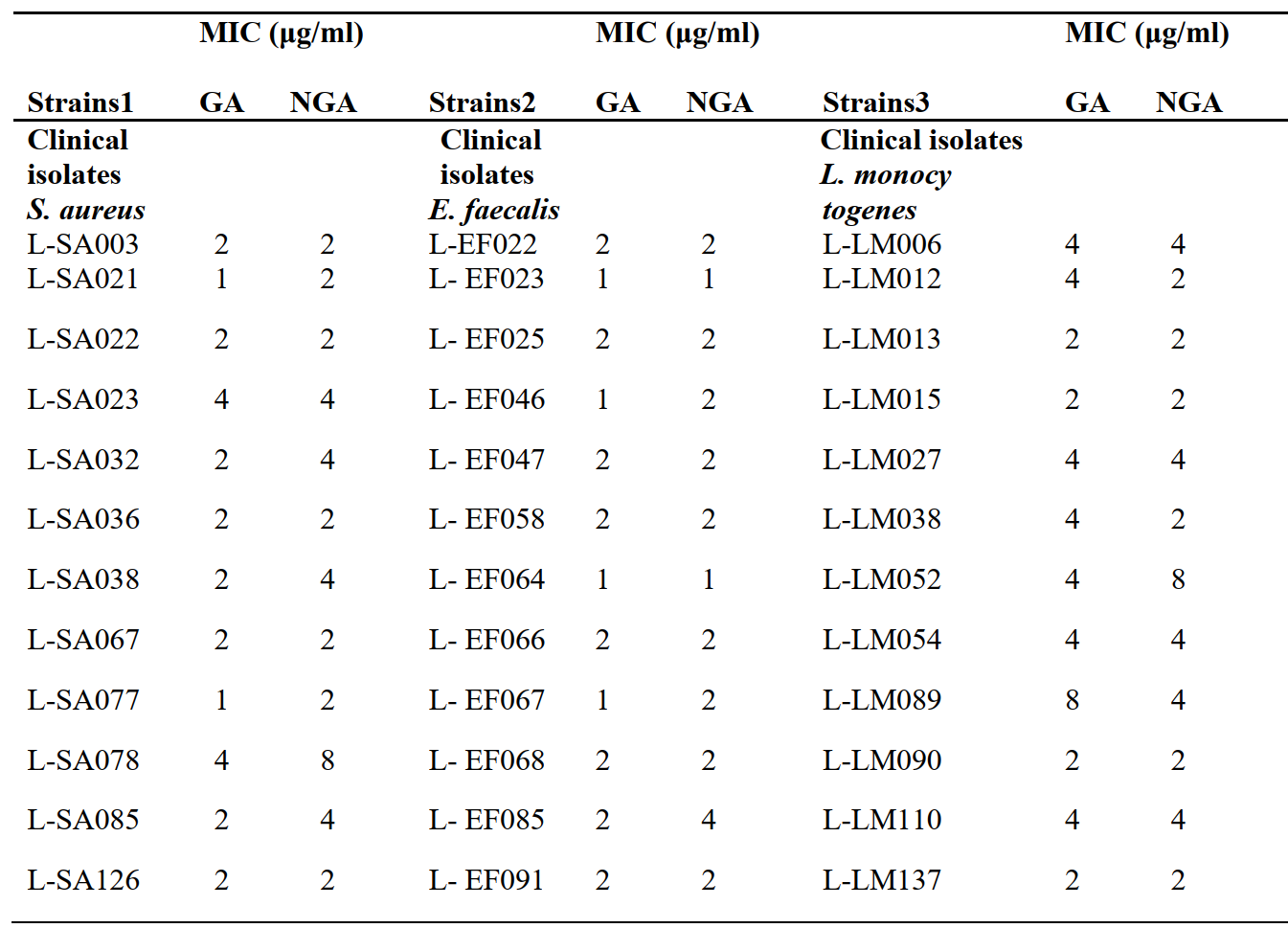


**Supplementary Figure S3.** MICs of GA and NGA against different clinical isolates. Bacterial cells were inoculated in LB broth for 18 h, and the MIC was determined to be the lowest concentration without visible bacterial growth. The data are presented as mean values from three independent biological replicates (*n*=3, SD=0).

**
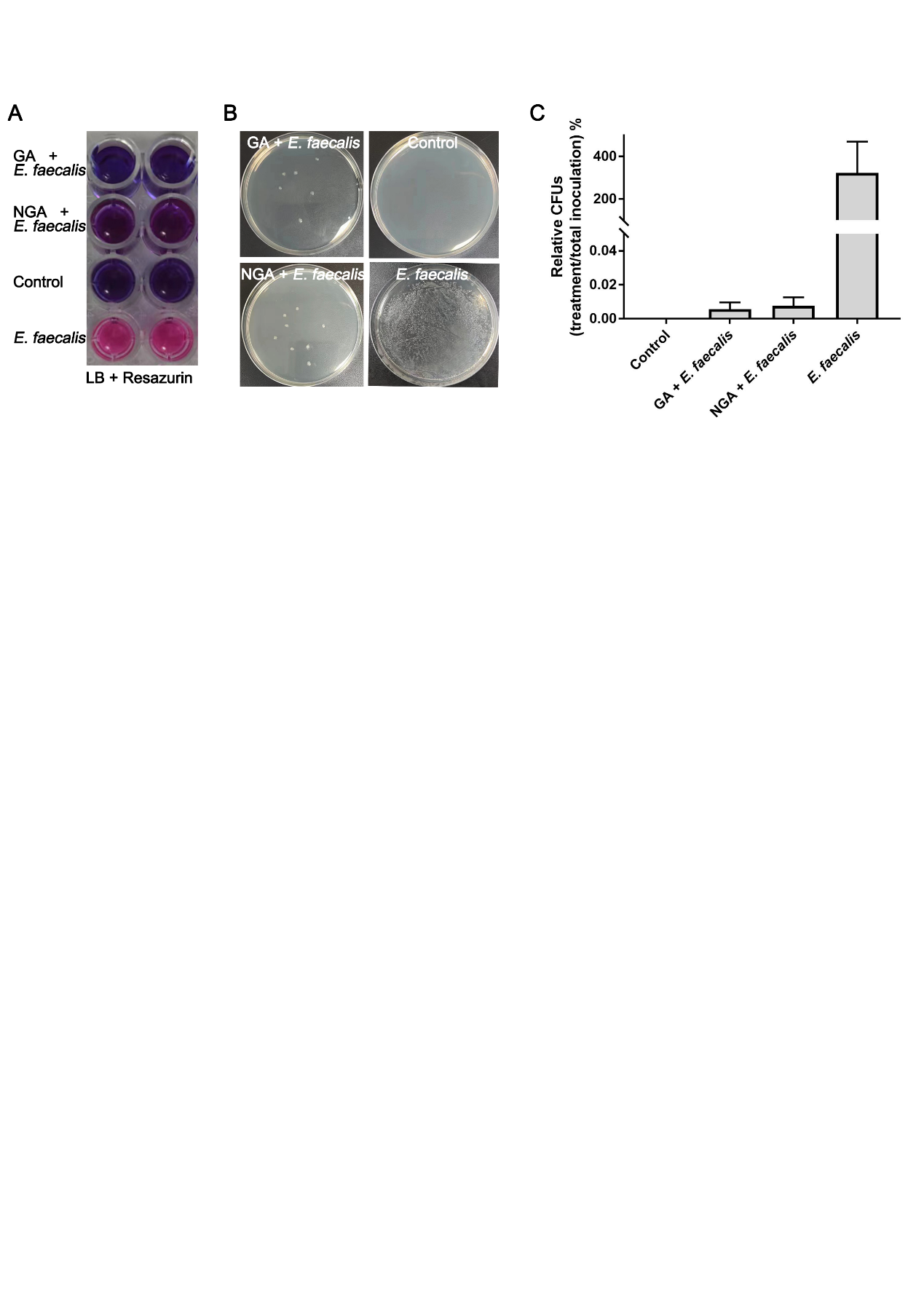
**

**Supplementary Figure 4.** The potential bactericidal properties for GA and NGA against *E.faecalis*. **(A)** The growth of *E. faecalis* in the presence/absence of 2 μg/mL GA or NGA in LB broth containing 0.1 mg/mL of Resazurin for 24 h. The LB broth was supplemented with 0.1 mg/mL of Resazurin only as a negative control. **(B)** The growth of *E. faecalis* on LB plates after 24 h of GA/NGA-treatment. **(C)** Quantification of the relative bacterial CFUs for panel **B**, the data are presented as the percentage of total bacterial CFUs after GA/NGA treatment *vs* total inoculations. The *E. faecalis* group counted by measuring OD_600_ before coated on the LB plate. The data are presented as mean ± SEM of three independent biological replicates.


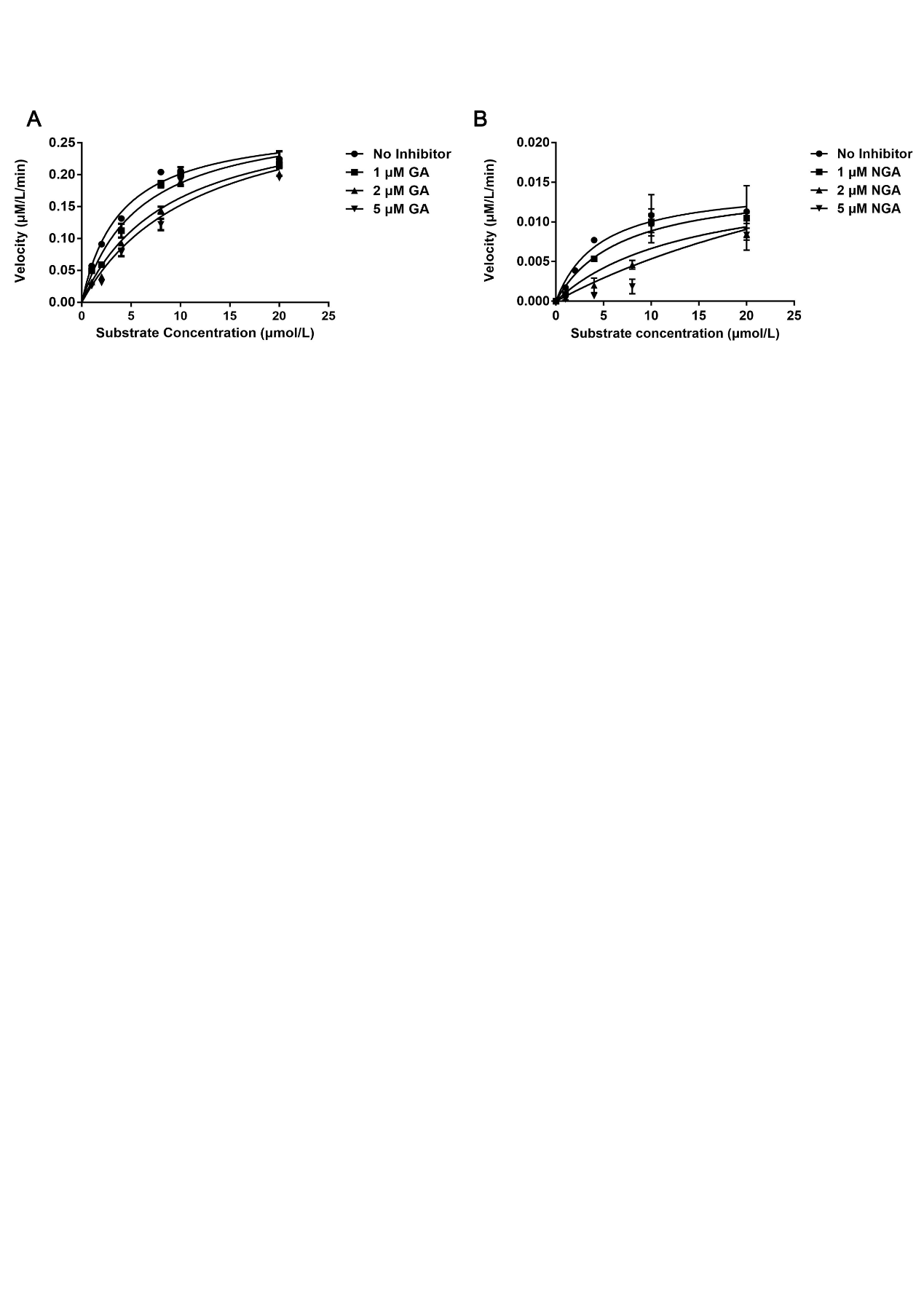


**Supplementary Figure S5.** Competitive inhibition by a range of concentrations GA or NGA of FPP binding to *Efa*UPPS. The enzyme activity experiment was performed in a reaction system containing 35 μM IPP, 0-20 μM FPP and 0.9 μM *Efa*UPPS, GA/NGA was added at the final concentration of 1/2/5 μM as the competitive inhibitors. The data are means of three independent biological replicates. Error bars represent mean ± SEM of *n*=3 experiments.


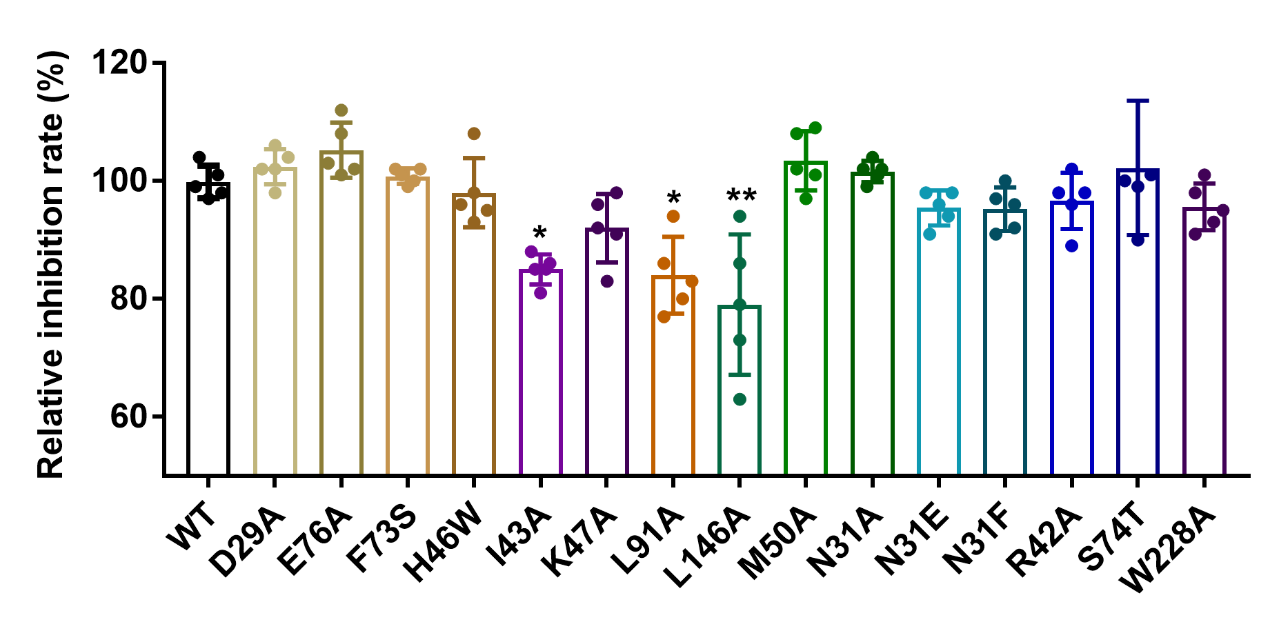


**Supplementary Figure S6.** Bar chart showing the *in vitro* inhibitory activities of GA against different *Efa*UPPS mutants compared with the activity of the wild-type *Efa*UPPS. The *Efa*UPPS inhibitory activity was performed by calculating the fluorescence percentage through determining the fluorescence absorbance of reaction mixture. The data are presented as the mean ± SEM of five independent biological replicates, and were analyzed by one-way ANOVA. (*, *P* < 0.05; **, *P*<0.01).


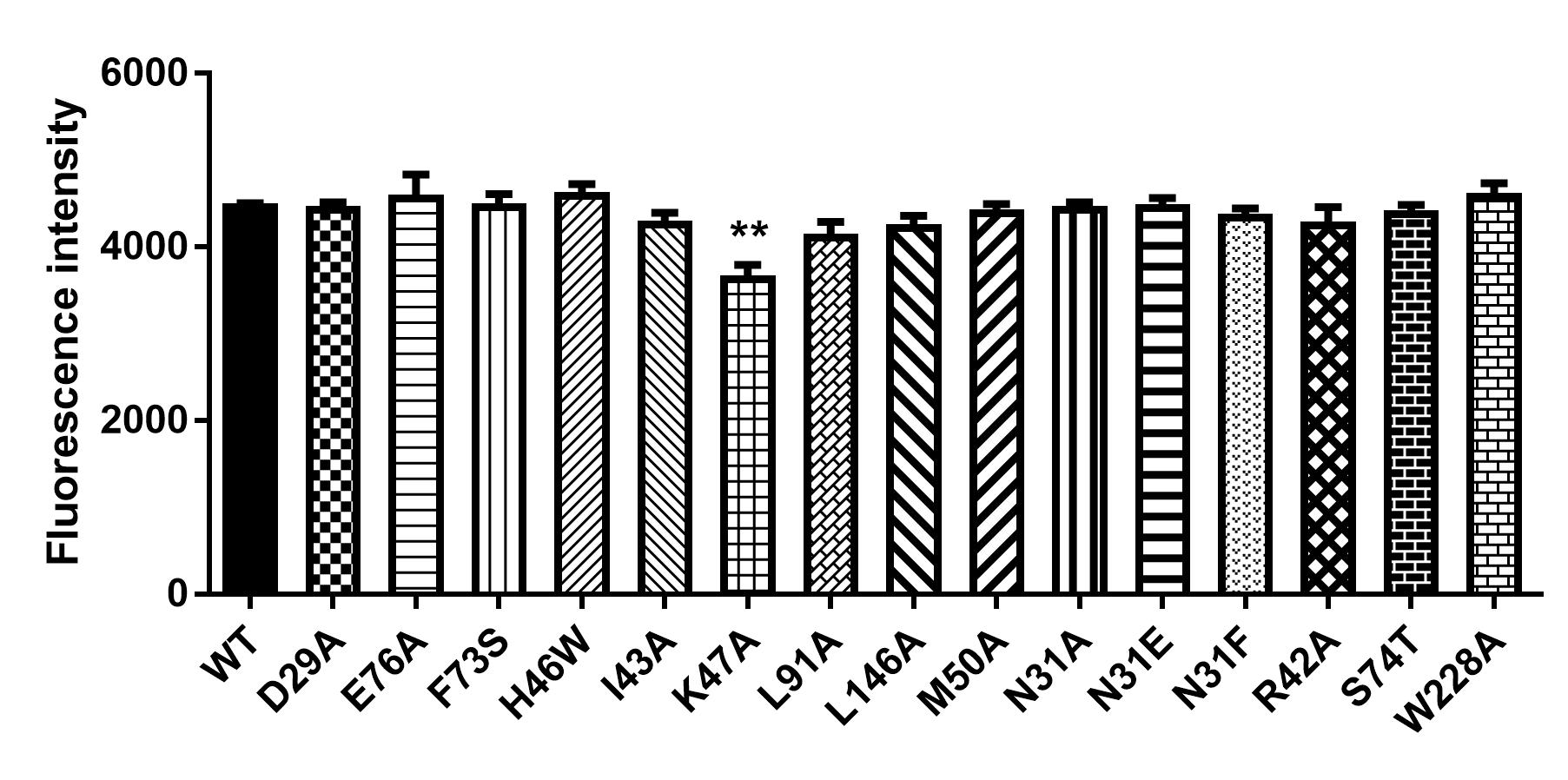


**Supplementary Figure S7.** Bar chart showing the enzyme activities of different *Efa*UPPS mutants compared with the wild type *Efa*UPPS. The figure indicated *Efa*UPPS enzyme activity was performed by determining the fluorescence absorbance of reaction mixture. The data are presented as the mean ± SEM of three independent biological replicates, and were analyzed by one-way ANOVA. (**, *P*<0.01).

*
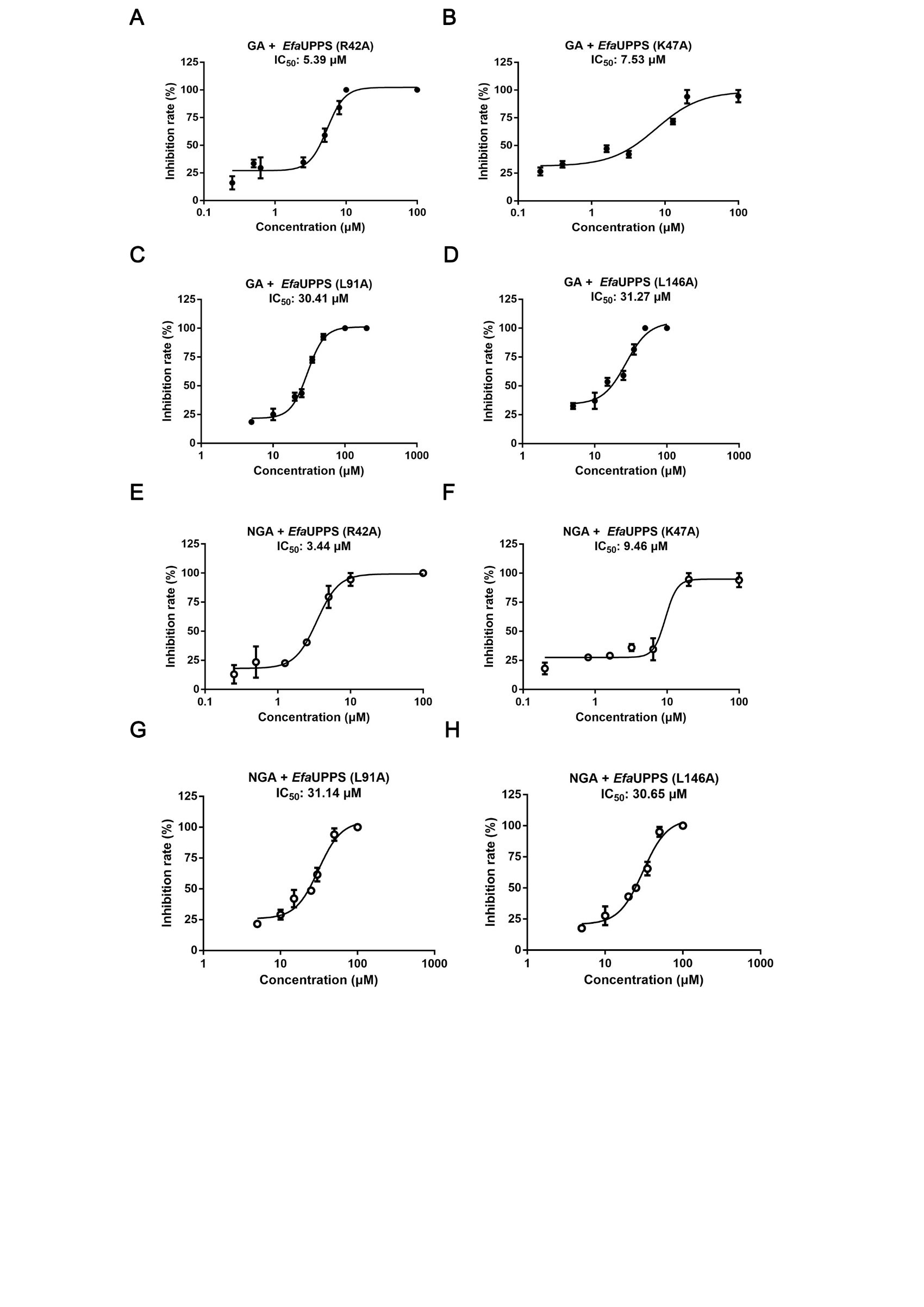
*

**Supplementary Figure S8.** *In vitro* inhibitory activities of GA **(A-D)** and NGA **(E-F)** toward different *Efa*UPPS mutants. The calculated fluorescence percentages were plotted *versus* GA/NGA concentrations on a semi-log scale (mean value ± SEM of three biological replicates). The IC_50_ was calculated under each curve. The results of GA were plotted with solid dots, while the results of NGA were plotted with hollow dots. *
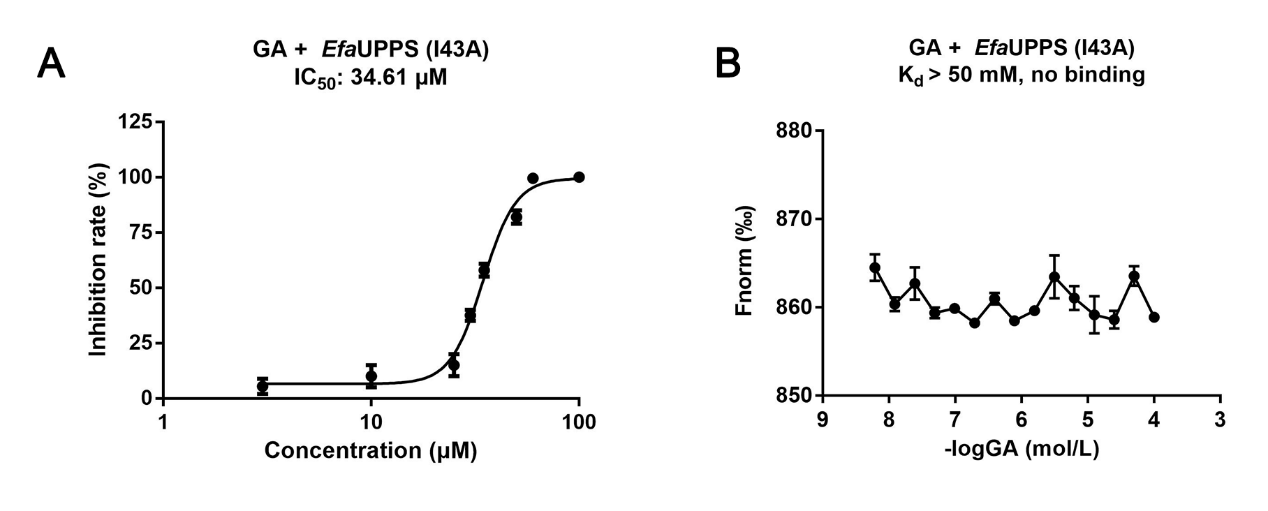
*

**Supplementary Figure S9.** The inhibitory activity **(A)** and binding affinity **(B)** of GA toward *Efa*UPPS I43A mutant. **(A)** The calculated fluorescence percentages were plotted *versus* GA concentrations on a semi-log scale (mean value ± SEM of three biological replicates). The IC_50_ was calculated under the curve. **(B)** The titration of GA ranged from 3.05 nM to 200 µM with a constant concentration of the *Efa*UPPS at 5 nM.


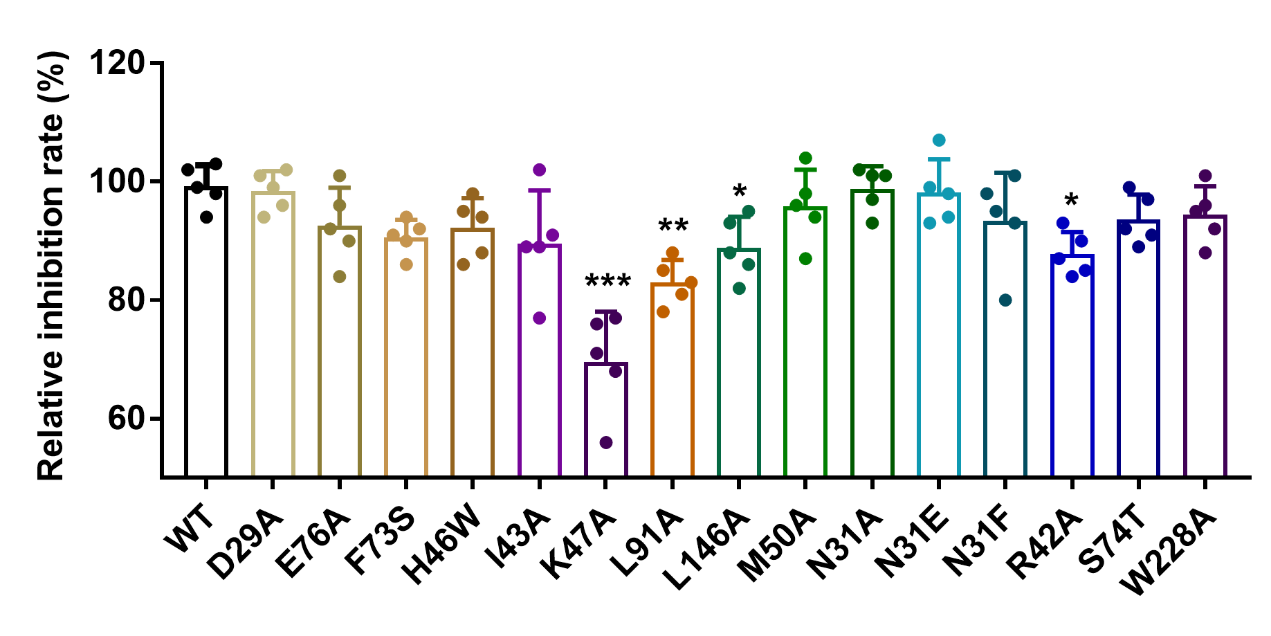


**Supplementary Figure S10.** Bar chart showing the *in vitro* inhibitory activities of NGA against different *Efa*UPPS mutants compared with the activity of the wild-type *Efa*UPPS. The *Efa*UPPS inhibitory activity was performed by calculating the fluorescence percentage through determining the fluorescence absorbance of reaction mixture. The data are presented as the mean ± SEM of five independent biological replicates, and were analyzed by one-way ANOVA. (*, *P* < 0.05; **, *P*<0.01; ***, *P*<0.001).


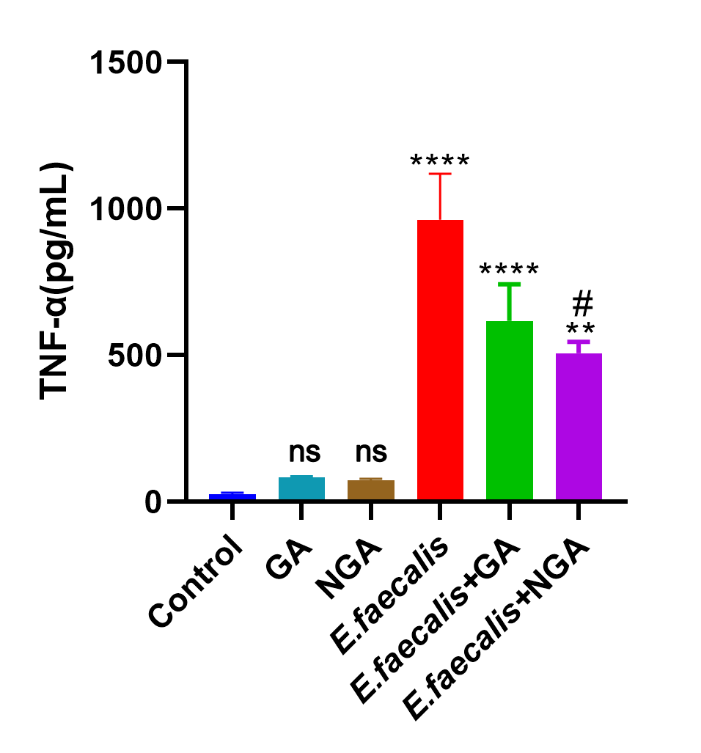


**Supplementary Figure S11.** The serum levels of TNF-α production in the different treated groups measured by ELSA. The data are presented as the mean ± SEM and were analyzed by ordinary one-way ANOVA with Tukey’s multiple comparisons. (** *P*＜0.01, **** *P*＜0.0001, ns, not significant, compared with control; # *P*＜0.05, compared with *E. faecalis* group.)


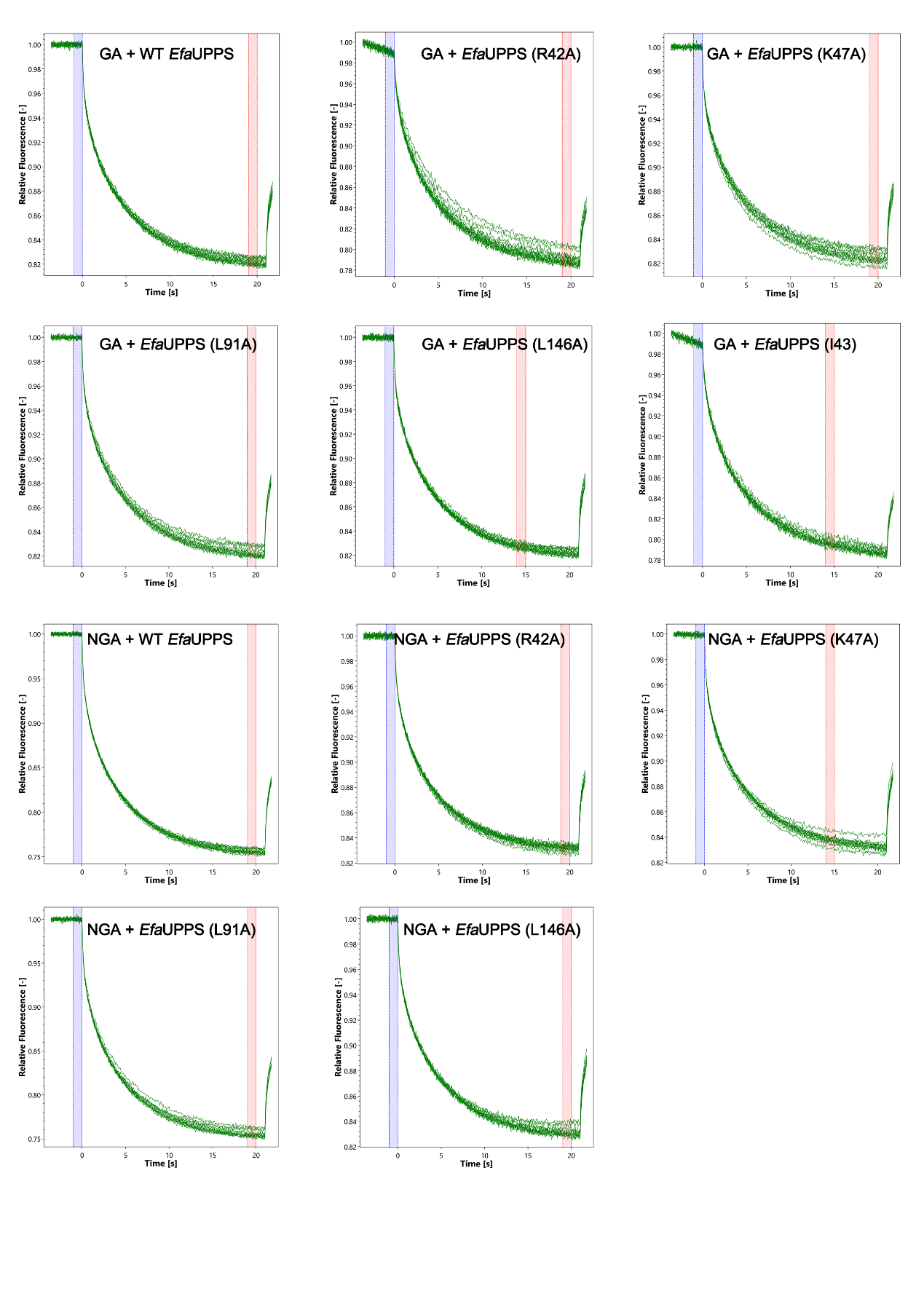


**Supplementary Figure S12.** The actual MST traces of GA and NGA toward the wild-type and different mutated *Efa*UPPS.
